# MAM kinases: physiological roles, related diseases, and therapeutic perspectives—a systematic review

**DOI:** 10.1186/s11658-025-00714-w

**Published:** 2025-03-28

**Authors:** A. Anjana Mohan, Priti Talwar

**Affiliations:** Apoptosis and Cell Survival Research Laboratory, 412G Pearl Research Park, Department of Biosciences, School of Biosciences and Technology, Vellore Institute of Technology, Vellore, Tamil Nadu 632014 India

**Keywords:** MAM, Kinases, ER stress, Mitophagy, Neurodegenerative disease, Cancer, Diabetes, Therapeutics

## Abstract

Mitochondria-associated membranes (MAMs) are tethering regions amid the membranes of the endoplasmic reticulum (ER) and mitochondria. They are a lipid raft-like structure occupied by various proteins that facilitates signal transduction between the two organelles. The MAM proteome participates in cellular functions such as calcium (Ca^2+^) homeostasis, lipid synthesis, ER stress, inflammation, autophagy, mitophagy, and apoptosis. The human kinome is a superfamily of homologous proteins consisting of 538 kinases. MAM-associated kinases participate in the aforementioned cellular functions and act as cell fate executors. Studies have proved the dysregulated kinase interactions in MAM as an etiology for various diseases including cancer, diabetes mellitus, neurodegenerative diseases, cardiovascular diseases (CVDs), and obesity. Several small kinase inhibitory molecules have been well explored as promising drug candidates in clinical trials with an accelerating impact in the field of precision medicine. This review narrates the physiological actions, pathophysiology, and therapeutic potential of MAM-associated kinases with recent updates in the field.

**Graphical Abstract:**

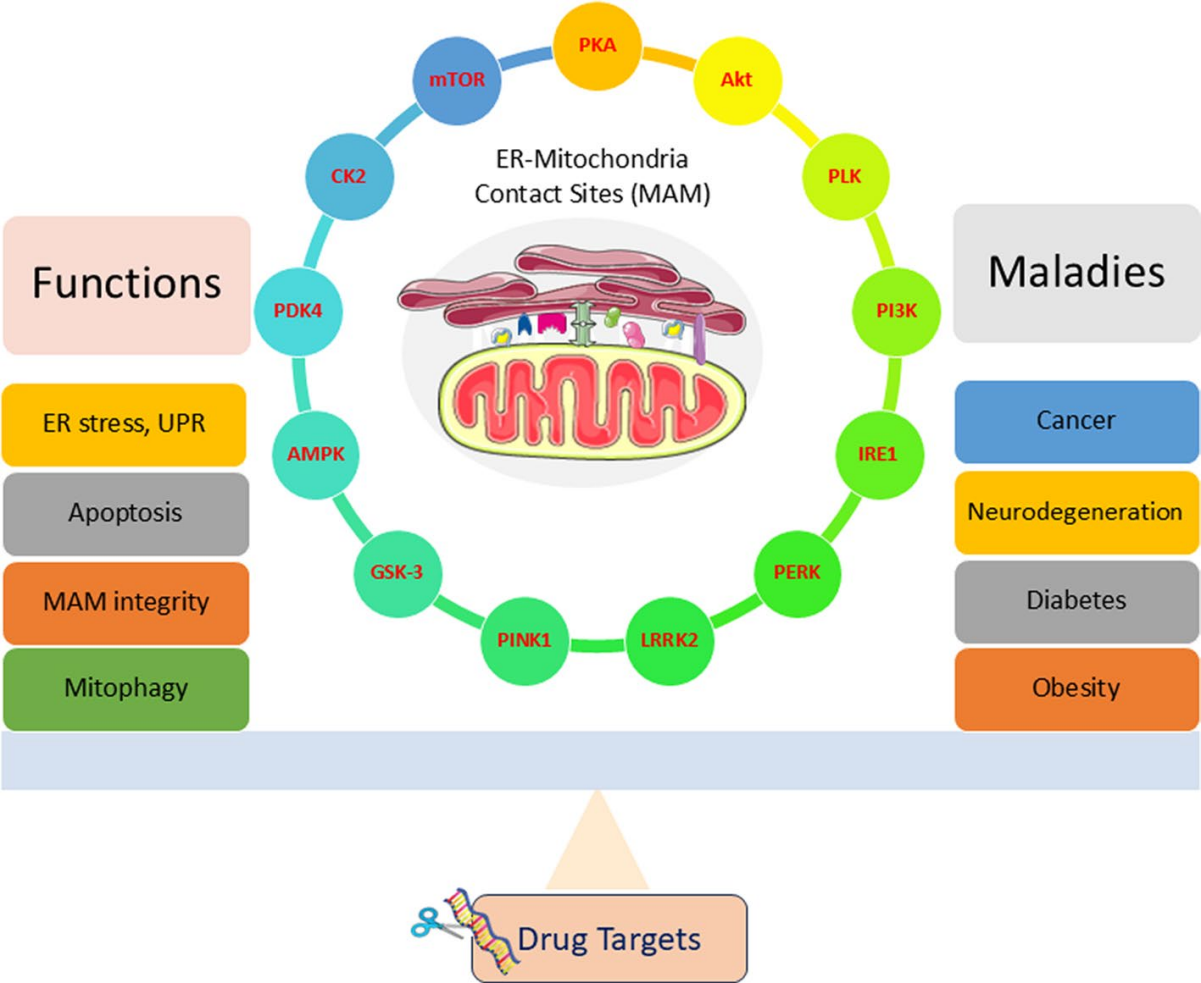

## Introduction

The presence of membrane-bound organelles characterizes eukaryotic cells. These organelles are interconnected with membranes; therefore their functions are controlled harmoniously. ER forms membrane contact sites (MCSs) and maintains a large network with most of the organelles, including the mitochondria [1, 2], Golgi apparatus, endosomes, peroxisomes, and plasma membrane (PM) [3]. Yeast cells are also not an exception, exhibiting the presence of the ER–mitochondria encounter structure (ERMES) complex [2] and are similar to an extent to MAM in higher trophic animals regarding protein composition and function [4]. The occurrence of a topographical relationship between ergastoplasm (rough endoplasmic reticulum) and mitochondria was revealed by some early electron micrograph studies conducted by Bernhard [5] in rat liver cells [5] and Copeland and Dalton [6] in cells of pseudobranch glands of a teleost, *Fundulus heteroclitus* [6]. In 1990, Vance described the presence of a membrane fraction “X” as an extension from mitochondria in crude rat liver tissue and its involvement in the synthesis and transport of various phospholipids. It is also inferred that the fraction “X” can assist with lipid trafficking between the two organelles, later becoming known as MAM [7]. MAM is formed by tethering about 15–20% of the outer mitochondrial membrane (OMM) to the smooth ER membrane [8, 9].

The dynamic nature of MAM facilitates the proximity adjustment between ER and mitochondria to meet various cytological demands. The MAM width ranges from 10 to 100 nm, and excessive closeness or separation may disrupt ER–mitochondria crosstalk and lead to abnormal conditions [10]. MAM serves as a platform for various protein–protein interactions and carries out metabolic activities such as energy homeostasis, lipid biosynthesis and transport, ER stress, antiviral responses [11], Ca^2+^ homeostasis [12], innate immune responses [13], and intracellular stress responses such as unfolded protein responses (UPR), autophagy, apoptosis, and inflammation [9]. Hence, dysregulation of MAM proteins underlies various clinical conditions, including neurodegenerative diseases [14], obesity, type 2 diabetes mellitus (T2DM) [15], cardiomyopathy [16], and cancer [17]. ER membrane proteins undergo posttranslational modification known as palmitoylation and tend to be enriched at MAM. Moreover, translocation of proteins from the cytoplasm and membranes makes the MAM a protein hub and crucial for various physiological activities. Regulation of MAM integrity and function is vital to maintaining the structure and functions of ER and mitochondria [18]. Mitofusin 2 (Mfn2) was the first protein to be recognized for ensuring ER morphology via proper ER–mitochondria contacts. Ablation of Mfn2 adversely affects ER structural features and mitochondrial Ca^2+^ uptake [19]. MAM-localized autophagosome formation is mandatory to remove defective mitochondria, while mitochondrial oxidative phosphorylation fails to correct ER stress [20]. Synthetic triterpenoid cyano enone of methyl boswellate (CEMB) can induce intrinsic, extrinsic, and ER stress-dependent apoptotic pathways in cancer cells, hence this compound can be evaluated as a potential cancer drug [21]. Several diseases including septic cardiomyopathy are reported to result from mitochondrial metabolic dysregulations such as defective mitophagy, bioenergetics, and ROS accumulation [22]. Alterations in mitochondrial biogenesis and respiratory chain pathways are observed as causative factors of lung malignancies. Dysfunctional mitochondrial quality control (MQC) results in altered mitochondrial fission, fusion, and mitophagy. Sappanone A is a plant-derived flavonoid that has proved effective in regaining mitochondrial biogenesis and bioenergetics, thereby impeding tumor progression [23]. These findings emphasize the importance of maintenance of lipid-raft-like ER subdomains (MAM) for the functional wellbeing of the cell and the whole organism [10].

Kinases can modulate functions and subcellular localization of targeted proteins by transferring γ-phosphate of ATP to the alcohol groups of amino acid residues such as serine (Ser) and threonine (Thr) and phenolic groups of tyrosine (Tyr) [24]. Human genome sequence analysis has counted around 560 different protein kinases, representing around 1.7% of human genes. Therefore, this is considered to be one of the largest protein superfamilies. Moreover, 60 atypical protein kinase families are present with kinase activities but without sequence similarities with the eukaryotic kinase domain [25, 26]. The general structure of a kinase enzyme consists of a C-lobe of α-helices and an N-lobe of β-sheets (β1–β5). Regulatory and catalytic spines are two hydrophobic spines on the α-helix that connect the C-lobe with the N-lobe. The catalytic spine is the binding site of ATP, and the regulatory spine denotes the active nature of kinase [27]. The catalytic domain or kinase domain consists of 250–300 amino acids and has subdomains with conserved residues, which provide the site of substrate binding and phosphoryl transfer. Phosphorylation of these residues is important for modulating the kinase activity. Ser/Thr kinases and Tyr kinases are two subdivisions of the kinase superfamily according to their phosphorylation sites [28, 29].

Several studies have been done regarding the effects of phosphorylation of MAM proteins and the resulting physiological events. Ninety potential kinase candidates were listed as MAM regulators in the MAM kinome library screening study conducted by Nhung et al. [9]. Any mutations or abnormal expression of kinases and related pathways may manifest as disorders such as malignancies, obesity, diabetes, neurodegeneration, and cardiomyopathy. Polo-like kinase 1 (PLK1) is a MAM kinase that regulates mitochondrial Ca^2+^ homeostasis, and its malfunctions have been well studied in various human malignancies [30]. An increased level of PLK2 was reported in brain tissues of patients with Alzheimer’s disease (AD) [31]. Likewise, several kinase candidates in the MAM subdomain play roles in pro-survival/pro-death pathways and can be considered to be dominant clinical targets in the twenty-first century [32]. The Food and Drug Administration (FDA) of the USA had approved 80 small-molecule kinase inhibitors as therapeutic agents as of January 2024. A permanent cure for degenerative diseases such as AD, Parkinson’s disease (PD), ALS, frontotemporal dementia (FTD), and prion disease is still a target for scientific society. A great deal of research has been done on kinases regarding their functions and relevance to health. However, many kinase candidates in MAM are yet to be discovered and discussed. Here, we provide an overarching understanding of the physiological actions, molecular aspects of pathogenesis, and therapeutic interventions of major MAM kinases, including available studies and updates. In addition, a brief consolidated discussion of MAM protein interactions is also provided. The review critically analyzes research gaps and reinforces further scientific exploration of the human kinome and its potential application for human wellbeing.

## MAM proteins: functions and malfunctions

MAM proteins are dynamic in their localization; they are mostly membrane-bound and are also found at the interface between the two organelles (Table 1). Del et al. listed 135 MAM proteins by immunoprecipitation of CHO cells containing amyloid β precursor protein (APP) mutation. Among these, 29% of the proteins were originally found in lysosomes, endosomes, cytosol, and plasma membranes other than ER or mitochondria [33]. A proteomics analysis performed by Sala-Vila et al. revealed an enriched abundance of the plasma membrane marker protein, flotillin. This is a liquid-ordered l_o_-domain marker that indicates the comparatively orderly packed region within the fluid membrane nature of MAM. Mass spectrometry analysis on MAM-enriched fractions resulted in a list of 1052 proteins [34]. The mass spectrometry proteome analysis of mammalian MAM conducted by Poston et al. reported 1212 high-confidence proteins [35]. Here, we present some of the MAM proteins and their crosstalk that is essential for cellular homeostasis.

**Table 1:** Summary of MAM proteins and their functions and related abnormalities

MAM protein	Interacting proteins		Functions	Cellular location	Related diseases	Refs.
Mfn2	Mfn1, Opa1		MAM tethering, MQC	ER membrane, OMM	Cisplatin-induced acute kidney disease, sepsis-associated intestinal injury, nonalcoholic steatohepatitis, NAFLD, T2DM, diabetic nephropathy, CMT2A	[19, 36, 37, 40, 43, 44]
TpMs	Mfn2		MAM anti-tethering, mitochondrial fragmentation	Mitochondria, cytoplasm, cytoskeleton, desmosomes	Breast cancer, colon cancer	[47–49, 229]
Fis 1	BAP31, Drp1		Apoptosis, mitochondrial fragmentation, mitophagy	OMM, peroxisome	AD, citrinin-caused intestinal damage	[51, 52, 54, 230]
PACS2	BAP31, calnexin		MAM integrity, movement of cargo proteins, Ca^2+^ flux	ER membrane, MAM	DEE66	[9, 56]
VAPB	PTPIP51		Ca^2+^ flux	ER membrane, MAM	ALS	[57]
IP3R	Grp-75, VDAC, Ero1α		Ca^2+^ flux, MAM tethering	Smooth ER, MAM, OMM	ALS, AD	[58, 62, 231]
FUNDC1	Drp1, Fis1, calnexin		Mitochondrial fragmentation, mitophagy	OMM	AD, PD	[64, 232]
Miga	Vap33		MAM formation	OMM, ER membrane	ALS	[74]
Cav-1	Mfn2, Drp1		Mitochondrial elongation	PM, MAM	Breast cancer	[162]
NLRP3	Undergo oligomerization		Inflammasome formation	MAM	Ulcerative colitis, depression, wound healing	[81–83]
PDZD8	Counter protein not known yet		MAM tethering, nonvesicular lipid transport	ER membrane	T2DM	[84, 85]
SYNJ2BP	E-Syt1, RRBP1		MAM tethering, Ca^2+^ flux	ER membrane	ALS	[84]

**Fig.1:**
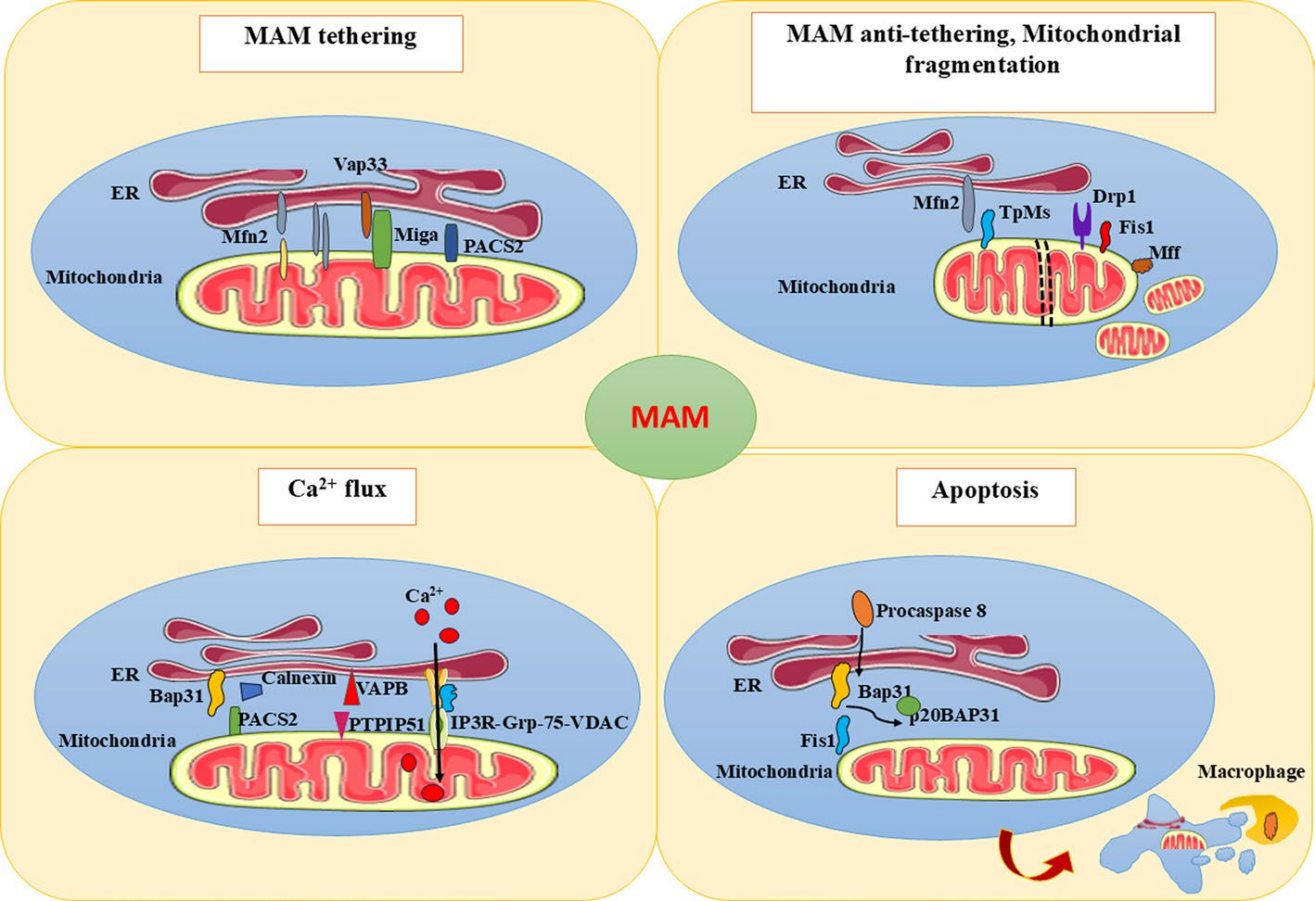
MAM-associated proteins and functions: MAM is a platform for anchoring various proteins that are crucial for ER–mitochondria (MT) tethering, mitochondrial fragmentation, Ca^2+^ homeostasis, and apoptosis

Mitofusin 2 (Mfn2) is a GTPase found in the OMM and ER membranes. Mitochondrial Mfn2 is engaged in fusion and regulates MQC [36], and ER Mfn2 enables bridging between two organelles by forming heterotypic or homotypic tethering complexes with Mfn1 and Mfn2 on OMM [19]. Mfn1 and Mfn2 are also involved in mitochondrial fusion tethering with another protein, optical atrophy 1 (Opa1) [37]. Splicing of Mfn2 produces two ER-specific variants: ER mitofusin 2 tether (ERMIT2) and ER mitofusin 2 (ERMIN2). ERMIT2 is involved in tethering and regulates ER stress and mitochondrial uptake of ER-released Ca^2+^. In contrast, ERMIN2 regulates ER morphology [38]. Intriguingly, Filadi et al. proved that ablation of Mfn2 increased ER–mitochondrial contacts, and such cells were highly sensitive to cell death signals owing to mitochondrial Ca^2+^ overload toxicity. Hence, they concluded that Mfn2 can act as a tethering antagonist to prevent toxic excessive proximity between two organelles [39] (Fig. 1). Stress signaling causes oligomerization of Mfn1 and 2 by disulfide bond formation at cysteine 684. Consequently, hyperfusion of mitochondria increases ATP production and serves as an adaptive stress response [36]. A recent study demonstrated that downregulation of Mfn2 resulted in reduced MAM integrity and increased mitochondrial reactive oxygen species (ROS), ER stress, and apoptosis in cisplatin-treated human proximal tubular cells. Hence, Mfn2 is a remarkable therapeutic target to address cisplatin-induced acute kidney disease [40]. Interestingly, downregulation of Mfn2 expression along with the application of essential oil from the plant *Chimonanthus nitens* Oliv. synergistically reduced MAM formation, ROS production, and NLRP3 inflammasome activation, which helped to mitigate lipopolysaccharide-induced sepsis-associated intestinal injury in rat models [41]. Exposure to cadmium chloride results in *S*-glutathionylation and mitochondrial-associated degradation of Mfn2. This inhibits Mfn2 from MAM localization, and the accumulation of necrosome complexes in the MAM causes neuronal necroptosis [42]. Deficiency and abnormalities in Mfn2 expression underlie the pathogenesis of non-alcoholic fatty liver disease (NAFLD), T2DM, diabetic nephropathy, and Charcot–Marie–Tooth disease type 2A (CMT2A) by loss of MAM integrity [43, 44]. Mfn1 and Mfn2 mediate OMM fusion, while Opa1 mediates inner mitochondrial membrane fusion. Alteration in the expression of these tethering proteins will reduce mitochondrial fusion and promote mitochondrial fission. These abnormalities can be causative factors for pulmonary arterial hypertension [45, 46].

Trichoplein/mitostatin (TpMs) is a keratin-binding protein in MAM that functions as an antitethering protein interacting with Mfn2 to encourage mitochondrial fragmentation. In turn, it reduces the Ca^2+^ flow from the ER to mitochondria and arrests apoptosis [47]. Increased levels of TpMs lead to decorin-tumor suppressor gene-mediated mitophagy in breast cancer cells. Mitostatin-induced mitophagy could be developed as clinical practice against cancers and diseases associated with mitochondrial dysfunctions [48, 49]. Mitochondrial fission, shape, morphology, and binding with ER are mediated by a mitochondrial transmembrane protein, fission protein 1 (Fis1) [50]. B-cell receptor-associated protein 31 (BAP31) at the ER membrane acts as a chaperone and interacts with Fis1. The interaction of Fis1 with BAP31 cleaves it into a pro-apoptotic fragment, p20BAP31. Following the signal, procaspase-8 is recruited to the Fis1–BAP31 complex depending on the availability of the variant of death effector domain in BAP31, which is necessary to activate procaspase-8. Consequently, Ca^2+^ flow from the ER to mitochondria occurs, resulting in cell death. The Fis1–BAP31 complex is responsible for the apoptotic signal and couples the two organelles to implement the same [51]. Increased oxidative stress, mitophagy, and lowered ATP levels were noticed in brain tissues from AD patients, resulting from increased interaction between Fis1 and dynamin-related protein 1 (Drp1) [52]. Fis-1-mediated Drp1 recruitment increases mitochondrial fragmentation and facilitates mitophagy. During hypercholesterolemia, Fis1 is translocated to the MAM interface, and the Fis1–BAP31 interaction results in ER–mitochondria contacts [53]. Intestinal damage is a major health problem caused by mycotoxin exposure (citrinin) in humans and animals. It results in ER–mitochondria proximity at a critical level due to the Fis1–BAP31 interaction [54]. The uncontrolled apoptosis in the intestine eventually disrupts the intestinal barrier. The alteration in the expression of proteins such as Mfn1, Mfn2, Fis1, and Drp1 results in a disturbance in mitochondrial fission and fusion. This will result in inadequacy of mitochondria, hindering metabolism and proliferation of vascular endothelial cells, and causing vascular injury, which may be an early event of many vascular-related diseases [55]. Phosphofurin acidic cluster sorting protein 2 (PACS2) is a multifunctional protein that maintains ER homeostasis and modulates the movement of cargo proteins between cellular compartments. It also mediates the function of BAP31 in maintaining MAM integrity, hence its depletion causes BAP31 fragmentation followed by apoptosis [56]. PACS2 recruits calnexin to ER–mitochondria contact sites and facilitates the Ca^2+^ flux between two organelles. The casein kinase 2 alpha 1 (CK2A1)-mediated phosphorylation of PACS2 facilitates the formation of a tripartite complex CK2A1–PACS2–PKD2 and mitochondrial Ca^2+^ influx via PKD2. Phospho-dead mutations in PACS2, such as E209K and E211K, ameliorate MAM integrity and lead to developmental and epileptic encephalopathy-66 (DEE66) [9].

Ca^2+^ oscillation between ER and mitochondria is carried out by another protein complex comprising vesicle-associated membrane protein-associated protein B (VAPB) at the ER and protein-tyrosine phosphatase-interacting protein-51 (PTPIP51) at the mitochondria. VAPB-P56S mutation is found to be a cause of type 8 ALS [57]. Inositol 1,4,5-triphosphate receptors (IP3Rs) are abundantly seen in the smooth ER and specifically function at the MCS between the ER and other organelles. The availability of Ca^2+^ at the MAM is ensured by a protein triad comprising IP3R type 1 IP3R1, glucose-regulated protein 75 (Grp75) molecular chaperone, and voltage-dependent anion channel 1 (VDAC1) at the OMM. Then, Ca^2+^ enters the mitochondrial lumen via the mitochondrial calcium uniporter (MCU) at the inner mitochondrial membrane (IMM) [58]. During insufficient Ca^2+^ storage in mitochondria, the Ca^2+^ flow through IP3R1–Grp75–VDAC1 is activated upon the detachment of binding immunoglobulin protein (BiP) from another Ca^2+^-sensitive chaperone, sigma-1 receptor (Sig-1R) [1]. Transglutaminase type 2 (TG2) is a key enzyme in MAM and interacts with Grp75. The lack of TG2–Grp75 interactions will result in enhanced IP3R–Grp75 signaling, reduced MAM contacts, and altered Ca^2+^ oscillation and MAM proteome [59]. VDAC1 is an OMM protein, alternatively known as mitochondrial pore protein, and acts as a gatekeeper protein that regulates the transit of metabolites across mitochondria [60]. Recently, the role of IP3R isoforms in maintaining the structural integrity of the MAM was explored. Each IP3R isoform is present in MAM, but IP3R1 is predominantly seen at ER–plasma membrane contact sites. Bartok et al. demonstrated that IP3R2 and IP3R3 are actively and efficiently involved in mitochondrial Ca^2+^ trafficking, and IP3R1 ensures sustained Ca^2+^ delivery to mitochondria. The study also proved the structural regulatory role of IP3R in MAM. The diminished contacts between ER and mitochondria in IP3R-lacking cells could be reversed by reexpressing any of the IP3R isoforms [61]. ER oxidoreductin 1α (Ero1α) is an abundant protein seen in MAM, interacts with IP3R, and is involved in ER Ca^2+^ and redox homeostasis [62]. IP3R2 interacts with another OMM protein, FUN14 domain containing protein 1 (FUNDC1), and ensures efficient MAM tethering and Ca^2+^ transfer. Deletion of FUNDC1 reduces the Fis1 expression and thereby mitochondrial fission and is a significant factor that leads to heart failure [63]. UNC-51-like kinase ULK1 activates FUNDC1 by phosphorylation at Ser-17 and results in mitophagy during hypoxic/ischemic conditions. During reperfusion, there is augmented phosphorylation at Ser-13 of FUNDC1 by casein kinase 2 (CK2), which reduces mitophagy [37]. Ablation of FUNDC1 causes diminished MAM contacts, reduced mitochondrial Ca^2+^ concentration, and mitochondrial Fis1 localization, eventually manifesting mitochondrial elongation. Under stress conditions, the interaction between FUNDC1 and calnexin became weak and new crosstalk between FUNDC1 and Drp1 builds up. Consequently, Drp1 is enrolled on the MAM and carries out mitochondrial fragmentation. FUNDC1 works in concert with Drp1, Fis1, or Calnexin to carry out mitochondrial division and mitophagy, hence abolition of any of these can result in mitochondrial imbalance [64]. Dynamin-related protein 1 (Drp1) is a mitochondrial fission protein that facilitates mitochondrial fission and is accelerated by the coupling between mitochondria and ER [65]. The phosphorylation activates Drp1 and is recruited to OMM by interacting with receptors such as Fis1 and mitochondrial fission factor (Mff). Drp1 becomes arranged into a ring-like structure around the mitochondrial membrane, divides into multiple mitochondria using GTP, and meets the cell requirements for additional energy [37]. Expression of Drp1 would be high in the heart under ischemic conditions. Conversely, Mfn2 and Opa1 will show diminished expression. This will lead to increased mitochondrial fission and ends up with apoptosis. FUNDC1-mediated mitophagy can protect cardiomyocytes from mitochondrial-induced apoptotic signaling. The excessive co-localization of Mff following myocardial ischemia/reperfusion (I/R) injury will result in pathological mitochondrial fission and induce a mitochondrial apoptotic pathway. Nuclear receptor subfamily 4 group A member 1 (NR4A1) is a member of the nuclear hormone receptor superfamily. Excessive expression of NR4A1 has been noted in the ischemic heart, and it causes Drp1-mediated mitochondrial fission. Wang et al. proved that NR4A1 is capable of exacerbating I/R injury by inducing abnormal Mff-mediated mitochondrial fragmentation, blocking FUNDC1-mediated mitophagy, and activating a mitochondrial pan-apoptotic pathway [66]. Receptor expression-enhancing protein-1 (REEP1) bends ER around mitochondria and promotes MAM contacts and Ca^2+^ transfer by interacting with VDAC1 [43]. Zishen Tongyang Huoxue decoction (TYHX) is a Chinese medicine used for treating sick sinus syndrome of sinoatrial node cells (SNCs). TYHX regulates VDAC1 expression and inhibits overmigration of Drp1 from the nucleus to the mitochondria. It reduces mitochondrial fission and improves mitochondrial dynamics, mitophagy, and mitochondrial membrane potential by stabilizing β-tubulin. TYHX offers protection against SNC injury upon hypoxia by ensuring MQC and reducing apoptotic signaling [67, 68]. Zishenhuoxue decoction (ZSHX) is another Chinese medicine that has vascular and myocardial protective effects. Applying ZSHX increased the expression of an anti-apoptotic factor TMBIM6 that inhibits VDAC1-mediated Ca^2+^ mitochondrial overload and ensures MQC [69]. Quercetin is found to be effective in providing myocardial protection following ischemia–reperfusion stress. Quercetin preserves MQC by mitigating mitochondrial oxidative stress and protecting cardiomyocytes [70]. Ginsenoside Rb 1 is a plant derivative that has been proven effective for the treatment of heart failure by regulating mitochondrial functions and regaining MQC [60].

A protein complex comprising Drp1, PINK1, and Parkin is formed at sites of mitochondrial damage and induces mitophagy [71]. Traumatic brain injury, AD, and status epilepticus are some of the neurological disorders that are characterized by elevated Drp1 activity, mitochondrial destruction, and inflammation [72]. Miga is an OMM protein that regulates ER–mitochondria tethering and autophagy [73]. The excessive interaction of Miga with ER protein Vap33 enhances MAM formation and leads to neurodegeneration and mutation in Vap33 in ALS [74]. The phosphorylation-dependent interaction of scaffolding protein caveolin-1 (Cav-1 Y14) inhibits the entry of Mfn2 and Drp1 to the mitochondria and the creation of the PINK1/Mfn2/Parkin complex in cancer cells. It halts mitochondrial fission/fusion and mitophagy [71]. Cav-1 is a PM-enriched protein and is vital for mechanosignaling and endocytosis. It translocates to MAM to regulate mitochondrial homeostasis and cholesterol efflux. Cav-1-knockout cells exhibited altered features such as unusual mitochondrial fragmentation instead of an elongated mitochondrial appearance, cholesterol accumulation in MAM, and depletion of steroid metabolism in MAM. Cav-1 mutations or deficiencies can lead to lipodystrophic conditions in humans [34]. MAM plays a significant role in the antiviral response signaling pathway carried out by a protein, mitochondrial antiviral signaling (MAVS), which activates transcription factors such as NF-κB and IRF3 as a part of the innate immune system [75]. MAVS is a transmembrane protein localized in the mitochondria, peroxisome, as well as MAM [11, 76]. Following the recruitment of MAVS to MAM, it interacts with another MAM protein, TRAF3, and evokes the innate immune system [13].

Notably, multiple regulators of the immune response could localize at the MAM to fulfill their activities, including the NLR family pyrin domain-containing protein 3 (NLRP3) inflammasome complex. NLRP3 is associated with mitochondria, and various stimuli such as mitochondrial Ca^2+^ overload, mitochondrial ROS accumulation, irremediable ER stress, and blocked mitophagy can activate the NLRP3 inflammasome [77]. NLRP3 is found to be associated with mitochondria and MAM during activation or overexpression of the NLRP3 inflammasome inducing inflammation signaling. NLRP3 triggers oligomerization of inflammasomes and recruits apoptosis-related proteins and cytokine secretion, facilitating robust immune responses [10]. NLRP3-mediated inflammation takes place in two steps: priming and activation. Priming is the transcriptional upregulation of NLRP3 expression by Toll-like receptor (TLR) signaling with agents such as lipopolysaccharides. The assemblage or oligomerization of NLRP3 into inflammasomes occurs during activation. The phosphorylation and dephosphorylation events are crucial for the activation of NLRP3 inflammasomes. The inflammasome activation is limited by IκB kinase (IKK) complex. c-Jun N-terminal kinase 1 (JNK1)-mediated phosphorylation at Ser194 of NLRP3 carries out priming. Phosphatase PP2a dephosphorylate NLRP3 at Ser5 and facilitates interaction between NLRP3 and an adaptor protein called ASC for inflammasome activation [78]. The increased diacylglycerol in the Golgi membrane recruits protein kinase D (PKD). NLRP3 at the MAM get activated and oligomerized upon PKD-mediated phosphorylation [79]. In resting cells, the inflammasomes are located in the ER, and during cellular imbalance it is redistributed around mitochondria and MAM. This destroys the mitochondrial structure and increases ROS production [80]. Licini et al. demonstrated the relevance of the ER–mitochondria crosstalk underlying NLRP3 inflammasome activation and its role in wound healing. They demonstrated delayed wound healing as a major side effect of the application of antibiotics using linezolid (LZD), an oxazolidinone antibiotic, in the case of methicillin-resistant *Staphylococcus aureus* (MRSA)-mediated bacterial skin infections. LZD mediates enhanced MAM tethering and interleukin-1β (IL-1β) production in keratinocytes, culminating in delayed wound closure. Diminished IL-1β production and accelerated wound repair were observed due to reduced ER–mitochondria contacts by downregulating PDZD8 [81]. ORMDL genes are encoded for ER transmembrane proteins and are risk factors in case of ulcerative colitis. Orosomucoid-like protein 3/ORMDL sphingolipid biosynthesis regulator 3 (*ORMDL3*) has specific roles in apoptosis, UPR, cytokine secretion, and inflammation. During ulcerative colitis, the concentration of ORMDL3 increases in MAM, augments ER–mitochondria tethering, and facilitates migration of NLRP3 inflammasomes from the ER to MAM and mitochondria, escalating IL-1β and IL-18 production. Moreover, the interaction between ORMLD3 and Fis1 increases, which induces mitochondrial fission. Downregulation of ORMLD3 can reduce inflammation and disease severity [82]. The interaction between extracellular ATP (eATP) and P2X7 receptors is capable of generating stress signaling. Stress induces Ca^2+^ trafficking via MAM modifications and IP3R3–Grp75–VDAC1 complex formation, eventually resulting in ER stress and mitochondrial damage. eATP supports NLRP3 aggregation in the MAM of hippocampal microglial cells and is a hallmark of depression-like symptoms [83].

PDZD8 is a MAM tethering protein with a synaptotagmin-like mitochondrial lipid-binding protein (SMP) domain and is involved in nonvesicular lipid transportation across the two organelles. PDZD8 is an ortholog of protein Mmm1 in yeast ERMES. Deficiency of PDZD8 negatively affects mitochondrial Ca^2+^ uptake [84] and neuronal Ca^2+^ dynamics [85]. The protein complex comprising SYNJ2BP on OMM and extended-synaptotagmin 1 (E-Syt1) on the ER membrane facilitates ER–mitochondria tethering and MAM formation, Ca^2+^ transfer, and mitochondrial lipid homeostasis. Overexpression of SYNJ2BP results in MAM formation and recruitment of E-Syt1 to mitochondria–ER contact sites (MERCSs). The proper mechanism behind SYNJ2BP and E-Syt1 interaction is not clear yet. SYNJ2BP also interacts with another ER protein, RRBP1, to regulate MAM integrity [84].

## Kinases: cell fate executioners in MAM

Phosphorylation and dephosphorylation are crucial events in signaling pathways. Kinases switch proteins on–off functional status. The MAM can be considered a hub of several proteins and metabolic pathways. Nhung et al. conducted MAM kinase screening and found 90 kinase candidates relevant to the DEE66 phenotype. Of these, 79 act as positive and 11 are candidates act as negative modulators of MAM. Among these kinase candidates, their roles in physiology and pathogenesis are known for only a few. Here, we present the structural details, general characteristics, main protein interaction, and pathways of known MAM kinases (Table 2).

**Table 2:** Summary of MAM kinases: targets, function, and associated diseases

Kinase	Target proteins	Functions	Diseases	Refs.
PKA	Drp1, Cav-1	MAM remodeling, Ca^2+^ flux, mitochondrial bioenergetics	NAFLD, breast cancer	[86–88, 159, 160, 162]
PKB	PACS2, IP3Rs, mTOR	Anti-apoptotic protein, blocks ER Ca^2+^ release	T2DM, ovarian cancer, breast cancer	[90, 91, 165, 166]
PLK	Miro	Regulates cell division, mitochondrial Ca^2+^ homeostasis	PD, carcinoma	[96, 98]
PI3K	Akt, Beclin-1	Pre-autophagosomal structure (PAS) formation	Rheumatoid arthritis, systemic lupus erythematosus, malignancies	[100, 102, 173]
IRE1α	CASP2, JNK	UPR, MQC, RIDD	Breast cancer, neuroblastoma	[104, 107, 109, 177, 179]
PERK	eIF2α, E-Syt1, MULAN, Parkin, MARCH5	Regulate ER–mitochondria contacts, regulate ER–PM contacts, UPR	Wolcott–Rallison syndrome, diabetic cardiomyopathy, AD, PD, dementia	[112–114, 118, 183]
LRRK2	SYNJ1, endophilin A1	Controls ER–mitochondria tethering	PD	[11, 233]
PINK1	Parkin, BECN1, Miro1	Regulates mitochondrial morphology, Ca^2+^ flux, complex I activity, ATP production, mitophagy, MQC	PD	[43, 127, 130, 190]
GSK-3	Glycogen synthase, MCL-1	Involved in apoptotic signaling	ALS, T2DM, FTD	[136, 137, 201, 203]
AMPK	ULK1	Mitophagy, autophagy	Diabetic nephropathy, myocardial hypertrophy	[138, 210, 212]
PDK	Pyruvate dehydrogenase, Grp75	Regulates MAM structure	Obesity, alcohol-associated liver disease	[145, 146, 216]
CK2	PACS2	Ca^2+^ transport over MAM	DEE66, AD	[9, 74]
mTOR	Akt	Regulate Ca^2+^ release at MAM, mitochondrial Ca^2+^ uptake	Diabetes, cancer	[91, 152, 219, 222]
MAPK	Drp1	Mitochondrial fission	Cancer	[225]

### Protein kinase A (PKA)

**Fig. 2:**
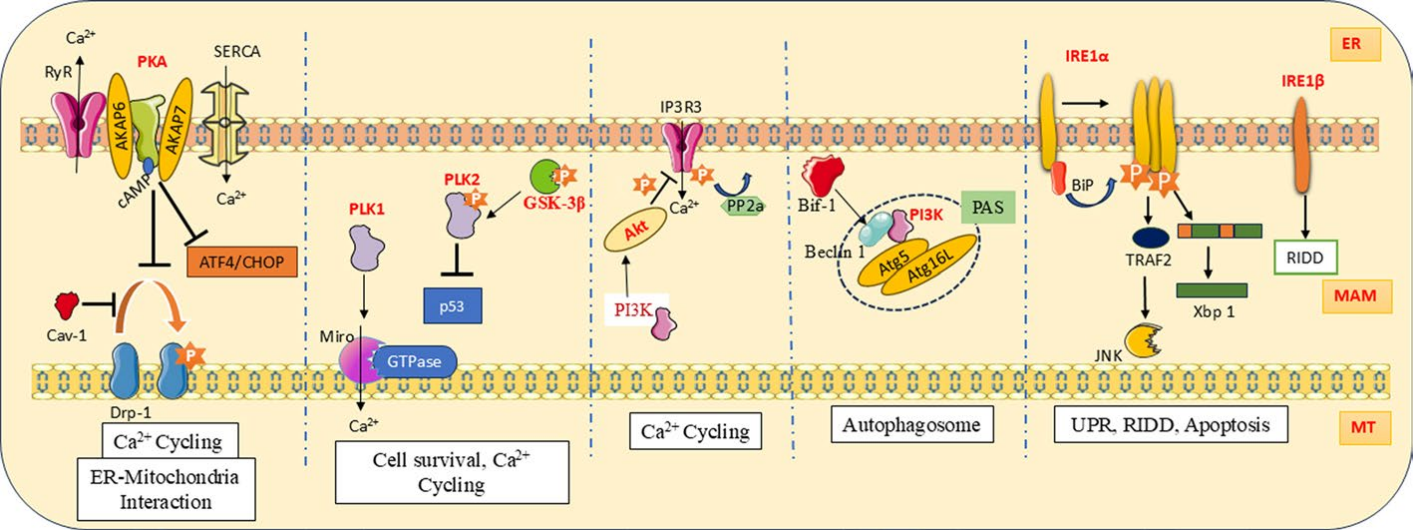
MAM kinases PKA, PLK, and Akt are involved in Ca^2+^ flux. PI3K forms a re-autophagosome structure (PAS) along with BECN1 and autophagosome markers. IRE1 is involved in stress responses such as UPR, RIDD, and apoptosis

The holoenzyme PKA comprises two regulatory units (R) and two catalytic units (C). There are two variants of regulatory subunits, namely R1 and R2, with four isoforms in action: R1α, R1β, R2α, and R2β. Cα, Cβ, and Cγ denote the three isoforms of catalytic subunits. AKAP6 and AKAP7 are the A-kinase anchoring proteins (AKAPs) that fasten PKA at the SR/ER and regulate Ca^2+^ cycling via ryanodine receptors (RyRs) and the sarcoplasmic/endoplasmic reticulum Ca ATPase-2 (SERCA) pump. PKA is pivotal in remodeling the contact between ER and mitochondria to promote organelle dynamics, Ca^2+^ flux, and mitochondrial bioenergetics during ER stress (Fig. 2) [86]. Drp1 S637 is the site of inhibitory phosphorylation by PKA during early ER stress and promotes mitochondrial elongation to cope with protein misfolding by impeding mitochondrial fragmentation and enhancing ER–mitochondria interaction. The presence of Cav-1 hinders PKA-mediated Drp1 phosphorylation and results in mitochondrial elongation, MAM reduction, and increased sensitivity to ER stress [87]. PKA and Cav-1 may work in a coordinated manner to ensure optimum ER–mitochondria coupling during ER stress. Activation of the cyclic adenosine monophosphate (cAMP)-dependent PKA pathway is considered a protective mechanism, as it inhibits ATF4/CHOP levels during prolonged ER stress [88]. PKA-mediated phosphorylation at Ser637 of Drp1 causes mitochondrial fission via the PINK1/Parkin pathway, which converges with multipolar spindle formation during the mitotic phase and induces mitotic defects and mitophagy [89].

### Protein kinase B (PKB/Akt)

The AGC kinase family comprises 63 different Ser/Thr kinases, including Akt or PKB, which is a known oncogene in mammalian cells. Three homologous classes are in action, namely Akt1/PKBα, Akt2 /PKBβ, and Akt3/PKBγ. Structurally, Akt has a pleckstrin homology domain at the N-terminal, a threonine-rich central kinase domain, and a serine-rich regulatory domain at the C-terminal [90]. Akt is activated by various growth factors and cytokines, with initial activation triggered by phosphoinositide 3-kinase (PI3K) (Fig. 2). Several proteins associated with apoptosis, proliferation, cell survival, differentiation, and metabolism are modulated by Akt-mediated phosphorylation. Akt phosphorylates PACS2 and takes part in stabilizing MAM [91]. All isoforms of IP3R possess a consensus sequence, RXRXX(S/T), in the cytosolic C terminal that is subjected to Akt-mediated phosphorylation [92]. Despite this, Akt inhibits ER Ca^2+^ release, mainly through IP3R3, and acts as an anti-apoptotic protein [93]. Besides, pro-apoptotic proteins such as procaspase-9 and BCL-2-antagonist of cell death (BAD) are inactivated by Akt [90]. Mice in Akt1^−/−^ condition showed shorter lifespans and increased spontaneous apoptosis upon genotoxic stress [94]. Phosphorylation of IP3R is reversed by PP2a and is impaired in cells devoid of a tumor suppressor, promyelocytic leukemia (PML). Hence, the Ca^2+^ flux through IP3R3 is suspended owing to the sustained kinase activity of Akt, and such cells show reduced cellular sensitivity to apoptotic signals [95]. Mammalian target of rapamycin (mTOR) kinase is activated by PI3K/Akt signaling and induces protein synthesis [90].

### Polo-like kinase (PLK)

PLK is a Ser/Thr kinase that regulates cell division and mitotic checkpoints. In mammalian cells, four homologs are found, namely PLK1, PLK2, PLK3, and PLK4. Among these, PLK1 is the prominent member involved in the onset of cell division and is found extensively in neoplastic tissues. Equilibrium of mitochondrial Ca^2+^ is known to be maintained by PLK-mediated phosphorylation of mitochondrial Rho GTPase Miro (Fig. 2). Miro is a component of mitochondrial transport machinery and interacts with Ca^2+^ transporters in the MAM. Following phosphorylation, the GTPase activity of Miro is induced and translocated to the MAM. The integrity of the MAM is ensured by the interaction of Miro with other MAM tethering complexes. Inactivation of Miro causes mitochondrial Ca^2+^ depletion, while overexpression leads to Ca^2+^ abundance in mitochondria and triggers apoptosis. The Polo–Miro axis is relevant in the self-renewal of neural stem cells and their differentiation by controlling mitochondrial Ca^2+^ homeostasis [96]. Oxidative stress causes activation of the PLK2/GSK-3β-mediated antioxidant signaling pathway. PLK2 supports the survival of cells with mitochondrial dysfunction by preventing p53-dependent necrotic cell death [97]. Treatment with protocatechuic aldehyde can induce overexpression of PLK, and thereby the activity of complex I, the mitochondrial membrane potential, and the level of free radicals are also increased; these effects have a positive impact on treatment for PD [98].

### Phosphoinositol 3-kinase complex (PI3K)

PI3K is a kinase family with three classes, namely class 1, class 2, and class 3, known for the phosphorylation of phosphatidylinositol lipids [99]. Beclin-1 interacts with class 3 PI3K (PI3KC3) and other autophagic proteins and facilitates their aggregation to form a pre-autophagosomal structure (PAS) (Fig. 2). Starvation leads to the expression of autophagic markers such as Atg5 and Atg16L via the activity of PI3KC3 [100]. The crosstalk between Beclin-1 and endophilin B1/Bax-interacting factor 1 (Bif-1) serves as an accelerating factor for PI3KC3 activation and induction of autophagy [101]. Mfn2 plays a pivotal role in maintaining the integrity of MAM during hypoxia and ischemic reperfusion injury. Mfn2 achieved the protective effect by activating the PI3K/Akt pathway and is capable of inducing anti-oxidant and anti-inflammatory effects in cardiomyocytes, thereby reducing mitochondrial damage and preventing myocardial apoptosis [102]. A study has demonstrated that restriction in calorie intake without malnutrition can induce autophagy in cortical neurons and resulted in anti-aging and reduction in the chance of age-related disorders. Caloric restriction and the hormone ghrelin induce autophagy via PI3K/Akt/mTOR inactivation [103].

### Inositol-requiring kinase 1 (IRE1α)

ER-residing IRE1 is a type I transmembrane protein kinase with three distinguished domains, namely an amino-terminus that projects toward the ER lumen and detects unfolded protein stress, a transmembrane helix, and a cytosolic C-terminal that initiates UPR with kinase and RNase activity [104]. It is involved in protein folding by activating chaperone genes and quality control to combat ER stress (Fig. 2). IRE1α and IRE1β are the two isoforms present in mammals. IRE1 is an inactive monomer and becomes an active oligomer owing to the detachment of BiP/GRP78 upon ER stress, and in turn, the RNase domain is activated by autophosphorylation [105]. IRE1α flag-off UPR either with the splicing of X-box binding protein 1 (Xbp1) mRNA or degrading of superfluous mRNAs via regulated IRE1-dependent decay (RIDD), which eventually ends up with apoptosis. The transcription of UPR-related genes for chaperones and apoptosis is accelerated by the entry of the Xbp-1 transcription factor into the nucleus [106].

In case of failure to achieve ER homeostasis, IRE1α signaling switches from adaptive responses and activates apoptosis. IRE1α can switch on programmed cell death through the upregulation of proapoptotic protease caspase-2 (CASP2) [107, 108]. IRE1α activates JNK with the help of an adapter protein, TNF receptor-associated factor 2 (TRAF2). IRE1α^−/−^ cells or cells with dominant negative TRAF2 cannot carry out JNK-mediated downstream apoptosis signaling [109]. Continuous kinase activation of IRE1α due to unresolved ER stress will lead to an incapability to respond to ER stress; instead, it will switch to apoptotic signals, while the pseudo kinase activation provides cytoprotection. The RNase activity of IRE1α mediates mitochondrial mRNA degradation and marks the onset of apoptosis [110]. Ubiquitination of IRE1α at lysin 481 by mitochondrial ubiquitin ligase (MITOL/MARCH5) at the MAM prevents hyperoligomerization, regulates RIDD, and inhibits ER stress-induced cell death. Mutation in IRE1α (K481R) or MITOL depletion reverses the aforementioned impacts [111]. Hence, IRE1 can be considered a “cell fate executor,” i.e., cell survival by UPR and RIDD, and cell death via apoptosis [105].

### RNA-dependent protein kinase (PKR)-like ER kinase (PERK)

**Fig.3:**
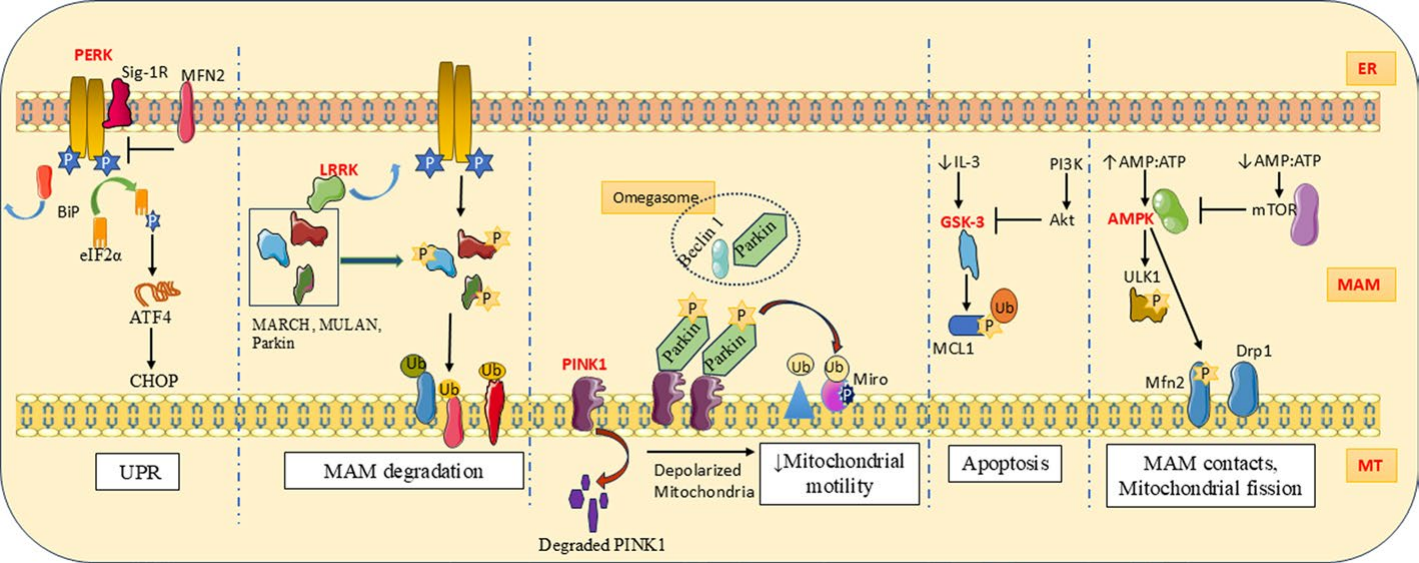
The MAM kinase PERK branch is one of the three UPR pathways. Mfn2 acts as the upstream regulator of PERK. LRRK detaches from E3 ubiquitin ligases and facilitates their PERK-mediated phosphorylation and subsequent MAM protein degradation. PINK1 actively participates in autophagy and modulates mitochondrial motility. GSK-3 regulates apoptosis, and AMPK regulates MAM contacts and fission according to the energy state of the cell

PERK/eukaryotic translation initiation factor 2 α kinase 3 (EIF2AK3) is a major sensor of ER stress and a vital component in the MAM as it facilitates ER–mitochondria juxtaposition. PERK is capable of modulating ER–mitochondria contact as well as ER–plasma membrane appositions [112] to propagate apoptotic signals to combat reactive oxygen species (ROS)-mediated stress, independently of its kinase activity in UPR [113]. Subcellular fractionation and western blotting in cardiomyocytes have proved the abundance of PERK in ER and MAMs coupled with Sig-1R (Fig. 3) [114]. Like IRE1, PERK is also an inactive monomer and becomes an active oligomer owing to the detachment of BiP due to ER stress. Sustained ER stress induces autophosphorylation and homo-multimerization of PERK. Consequently, the translation of all the genes except ATF4 is suspended owing to PERK-mediated phosphorylation of the eukaryotic transcriptional initiation factor-α subunit (eIF2α). The apoptosis occurs owing to the upregulated manifestation of pro-apoptotic C/EBP homologous protein (CHOP) by ATF4. PERK is the key ER stress sensor that can induce activation of CHOP [107]. PERK functions as an ER stress sensor and MAM tethering modulator by phosphorylating and activating E3 ubiquitin ligases [115]. Interactome analysis revealed that PERK moonlights at MAM and aids Ca^2+^ signaling by interacting with the SERCA pump [116]. PERK^−/−^ cells show disrupted ER morphology, weaker ERMCS, and Ca^2+^ signaling. PERK^−/−^ murine embryonic fibroblasts (MEFs), showed reduced sensitivity to apoptosis following ROS signaling between ER and mitochondria. Hence, PERK-deficient cells are vulnerable to apoptotic defects during unfavorable conditions associated with ROS-mediated ER stress [113]. In healthy cells, Mfn2 keeps PERK in an inactive state, but the high glucose level resulted in Mfn2 reduction and activation of PERK. In turn, this resulted in a disturbance in the MAM and mitochondrial dysfunction. Muñoz et al. established that Mfn2 can act as an upstream modulator of PERK and Mfn2 ablation causes sustained kinase activation. In turn, there will be a heightened UPR and reduced tendency for apoptosis [117]. Apart from UPR, PERK moonlights to facilitate phospholipid transportation across the ER and mitochondria. Diminished expression of PERK results in a reduced MAM area and MAM-mediated phospholipid transportation. E-Syt1 is a lipid-transferring protein that tethers the ER with the plasma membrane (PM) and peroxisome. Higher intracellular Ca^2+^ concentration promotes the binding of the C2C domain of E-Syt1 with PI(4,5)P2 in the PM. E-Syt1 is a prospective interactor of PERK, and PERK recruits the protein to the MAM by binding on its C2D and C2E domains. It is observed that the PERK/E-Syt1 axis is necessary for mitochondrial lipid homeostasis and thereby mitochondrial respiration. The ameliorated lipid trafficking due to mutations in PERK and possibly the lack of PERK/E-Syt1 interaction is manifested as Wolcott–Rallison syndrome with aberrant lipid metabolism and mitochondrial dysfunctions [118].

### Leucine-rich repeat kinase 2 (LRRK2)

LRRK is a member of the ROCO protein family with two homologs: LRRK1 and LRRK2. Familial PD is a result of LRRK2 gain-of-function mutation, and loss-of-function mutation in LRRK1 results in bone diseases [119]. LRRK2 controls ER–mitochondria tethering through PERK-mediated phosphorylation. Kinase active LRRK2 (G2019S) gets separated from the E3 ubiquitin ligases such as MULAN, Parkin, and MARCH5 and becomes susceptible to PERK-mediated phosphorylation and activation (Fig. 3). Subsequently, the MAM proteins get ubiquitinated and become susceptible to degradation. Meanwhile, the kinase‐dead mutation (D1994A) of LRRK2 makes it constitutively bound to these ligases, avoiding PERK‐mediated activation of ligases, hence ER–mitochondrial tethering proteins remain functional and increase ER–mitochondria contacts [115]. The mutation affects the GTPase and kinase activity of LRRK2. LRRK2–G2019S mutation results in enhanced ER–mitochondria associations, SERCA inhibition by sustained interaction with phospholamban (a regulatory protein in ER), mitochondrial Ca^2+^ overload, ER Ca^2+^ depletion, and eventually mitochondrial dysfunction [120].

### PTEN-induced putative kinase 1 (PINK1)

Ser/Thr kinase PINK1 regulates mitochondrial morphology, Ca^2+^ trafficking, complex I activity, ATP production, mitophagy, and the quantity and quality of mitochondria [43]. A gene (*PARK6*) comprising 8 exons with 581 amino acids codes for PINK1 [121]. Structurally, it possesses a mitochondrial targeting sequence at the N-terminus and a kinase property at the C-terminus [122]. Meanwhile, mature PINK1 has been found in both mitochondria and cytosol [123]. Under basal conditions, mitochondrial processing peptidase (MPP) recruits PINK1 to the IMM from the OMM and is cleaved by mitochondrial proteases [124]. PINK1 translocation and degradation take place depending on the mitochondrial membrane potential [125]. Narendra et al. proved that, upon mitochondrial uncoupling or damage, the membrane potential decreases and the ubiquitin ligase Parkin is translocated from the cytosol to the mitochondrial membrane [126]. Hence, depolarized mitochondria stabilize PINK1 on OMM and recruit Parkin. Parkin is a downstream substrate of PINK1 [127], and mitophagy of superfluous mitochondria is induced by activated Parkin [126]. Alterations in PINK1 exacerbate the mitochondrial membrane potential by affecting complex I activity [128]. A study in SH-SY5Y cells reported that, during mitophagy, Parkin and pro-apoptotic protein Beclin-1/BECN1 are assembled in the MAM, and subsequent interaction between PINK1 and BECN1 induces the formation of ER–mitochondria contacts and omegasomes, a precursor form of autophagosomes (Fig. 3) [129]. It is observed that the rate of anterograde and retrograde motility of mitochondria in the neurites of differentiated cells was diminished in PINK1-knockdown cells. Lack of functional PINK1 resulted in various effects including increased inhibitory phosphorylation of GSK‐3β at Ser9, increased release of mitochondrial Ca^2+^ when exposed to carbonyl cyanide *m*-chlorophenyl hydrazone (CCCP)‐induced mitochondrial uncoupling, and reduced ER–mitochondria contacts [123].

The motor adaptor protein (mitochondria anchoring protein) Miro1, also known as RhoT1 or RhoT2, is a substrate of PINK1 and undergoes phosphorylation followed by ubiquitination. Mimicking PINK1 phosphorylation of Miro on S156 recruited Parkin, induced mitochondrial fragmentation, and mitochondrial transport arrest, but was not efficient enough to complete the mitophagic pathway without PINK1. The same with Miro T298/299 did not result in the above-mentioned effects. Hence, it was deduced that the phosphorylation site on Miro would regulate the subsequent effects led by Parkin [130]. Parkin is recruited on phosphorylated Miro and triggers Miro degradation, which in turn causes declined mitochondrial motility. This event marked the onset of mitophagy [131]. A study by Pei-I Tsai et al. on *Drosophila* Miro (DMiro) protein showed the neuroprotective role of PINK1. DMiro with phospho-dead mutation protected DMiro from PINK1-mediated phosphorylation and further degradation. This resulted in increased mitochondrial transport and loss of synaptic integrity, especially dopaminergic neurons [132]. Mitochondrial degradation via mitophagy was found to be accelerated by the dissociation of Mfn2 from OMM due to PINK1/Parkin-mediated phosphorylation and a p97-dependent pathway. This negatively affects the structure and function of MAM [133].

### Glycogen synthase kinase-3 (GSK-3)

Glycogen synthase kinase‐3 (GSK-3) takes part in physiological functions such as apoptosis, membrane permeability, and bioenergetics [134]. GSK-3 is recognized for its role in regulating glycogen synthesis in response to insulin. Glycogen synthase is the substrate of GSK3 [135]. There are two homologous isomers for the enzyme GSK3, namely GSK-3α and GSK-3β, with different functions. Unlike other kinases, Akt-mediated phosphorylation of Ser-21 and Ser-9 causes the suppression of GSK-3α and GSK-3β, respectively [136]. PI3K-Akt-mediated phosphorylation of GSK-3β is cardioprotective during ischemic preconditioning. The same cytoprotective effects can be granted by applying GSK-3β inhibitors such as lithium and SB 216763. GSK-3β inhibition can offer better post-ischemic functions and cell survival by reducing the chance of apoptosis [135]. Cellular Ca^2+^ overload is the main factor that leads to reperfusion injury-dependent cell death. A fraction of cellular GSK-3β is always present in the ER–mitochondria interface and interacts with the protein complex comprising IP3R, VDAC1, Grp76, and cyclophilin D. IP3R-mediated Ca^2+^ release from SR to mitochondria was mitigated following GSK-3β inhibition and provided cytoprotection by avoiding cellular and mitochondrial Ca^2+^ overload. Hence, GSK-3β can be considered a target to improve the efficacy of cardioprotection against reperfusion injury. Whether the IP3R is a direct downstream target of GSK-3β is yet to be clarified [136]. Under low concentrations of growth factors, the anti-apoptotic protein MCL-1 is phosphorylated by GSK-3β and induces apoptosis. This is followed by ubiquitin-mediated proteasomal degradation. PI3K/Akt signaling can protect apoptotic signals by phosphorylated inhibition of GSK-3β. A study conducted in FL5.12 cells upon withdrawal of IL-3 proved the role of the PI3K/Akt/GSK-3β/MCL-1 cascade in cell survival and cell death of neuronal and hematopoietic cells. Ablation of GSK-3β negatively affects mitochondrial membrane permeability [137].

### Adenosine monophosphate-activated protein kinase (AMPK)

AMPK can sense energy levels in cells and fine-tune cytological functions accordingly to ensure energy homeostasis. There is a catalytic subunit (AMPKα) and two regulatory subunits (AMPKβ and AMPKγ) in the holoenzyme. AMPK can be activated under increased AMP:ATP ratio, glucose deprivation, and cell energy stress. ULK1 undergoes AMPK-mediated phosphorylation in response to hypoxia and glucose starvation (Fig. 3). In turn, ULK1 is translocated on dysfunctional mitochondria and is involved in mitophagy/autophagy [138]. Another study revealed that loss of ER–membrane protein BAP31 can induce autophagy by activating the AMPK–ULK axis. At the same time, a raised level of phosphorylated AMPK indicates ATP depletion in BAP31-knockout cells [139]. AMPK enters mitochondria and the MAM during energy stress and phosphorylates Mfn2. The AMPK–Mfn2 axis increases MAM contacts, mitochondrial fission, and autophagy [140]. Under nutrient sufficiency, mTOR inhibits activation of ULK1 by disrupting the interaction between ULK1 and AMPK and avoiding autophagy [141]. AMPK phosphorylates Mff, an OMM receptor for Drp1, and induces mitochondrial fragmentation. Rapid AMPK-dependent induction of mitochondrial fission following prolonged mitochondrial stress prepares cells to initiate mitophagy and maintains mitochondrial homeostasis [142]. How AMPK regulates both MAM formation and mitophagy during stress conditions remains unclear. AMPK can act as an anti-inflammatory factor by reducing ROS production. Wen et al. demonstrated that the saturated fatty acid palmitate causes AMPK inhibition. It further prompted dysregulated autophagy of damaged mitochondria and increased ROS production, both of which lead to activation of the NLRP3 inflammasome. It assembles on the ER–mitochondria complex and resulted in inflammation. This may be how mitochondrial damage leads to inflammatory diseases [143, 144].

### Pyruvate dehydrogenase kinase (PDK)

**Fig.4:**
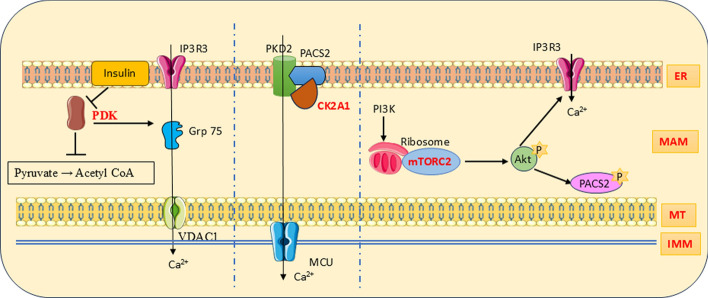
MAM kinase PDK is involved in Ca^2+^ transport via the IP3R–Grp75–VDAC1 channel and inhibits acetyl CoA formation from pyruvate. The activity of PDK is regulated by insulin. CK2A1 takes part in Ca^2+^ transport via PKD2. Ribosome-attached mTORC2 phosphorylates Akt and carries out downstream events

PDKs are a type of mitochondrial matrix enzyme capable of phosphorylating pyruvate dehydrogenase. Consequently, the conversion of pyruvic acid to acetyl CoA is suppressed (Fig. 4). Among four isoforms (PDK1–4), mammalian skeletal muscles and cardiomyocytes have PDK4 abundantly. PDK4 interacts with the IP3R1–GRP75–VDAC1 axis and regulates MAM structure and functions. Individuals suffering from obesity are also susceptible to mitochondrial Ca^2+^ overload, associated dysfunctions, ER stress, and insulin resistance. These effects are due to increased PDK4 activity and corresponding MAM formation. Mouse models with diet-induced hyperglycemia showed a decline in MAM levels and insulin resistance on ablation of PDK4 [145]. The abundance of PDK4 mRNA is suppressed by insulin. The presence of circulating free fatty acids such as oleic acid and palmitic acid can attenuate the inhibitory effect of insulin on PDK4. This indicates the high concentration of PDK4 and its abnormal manifestations in obese individuals [146].

### Casein kinase II (CK2)

CK2 is a Ser/Thr protein kinase with heterotetrameric structure with two catalytic subunits, namely CK2α and CK2α′, and a dimerized regulatory subunit CK2β [147]. It plays regulatory functions in mitochondrial equilibrium, mitochondrial autophagy, cell death, intracellular Ca^2+^ homeostasis, and mitochondrial fusion. The MAM structure and Ca^2+^ crosstalk over the MAM are found to be regulated by the catalytic subunit of CK2 (CK2A1) via the CK2A1–PACS2–PKD2 cascade (Fig. 4) [9]. During mitophagy, the OMM protein Atg32 is phosphorylated and interacts with Atg11. CK2 is essential for Atg32 phosphorylation at serine 114 and serine 119, Atg32–Atg11 interaction, and mitophagy [148]. Notably, CK2 uses both ATP and GTP for phosphorylation purposes. Unlike other enzymes, CK2 is active irrespective of its monomeric and tetrameric forms and the availability of any cofactors. As CK2 is a potent inhibitor of cell death, its dysregulation leads to malignancies [147].

### Mammalian target of rapamycin (mTOR) kinase

mTOR is a nutrient-sensing atypical Ser/Thr kinase complex known for regulating cellular functions such as metabolism, division, and growth in response to growth stimuli and nutrients such as glucose and amino acids [149]. mTOR complex 1 (mTORC1) and mTOR complex 2 (mTORC2) are two isoforms in mammalian cells. mTORC2 is profoundly seen in the MAM in the ribosome-bounded form (Fig. 4) [150]. mTORC1 plays a major role in protein translation with a unique feature of specific binding with rapamycin [151]. Growth factor-dependent MAM localization of mTORC2 facilitates the interaction with IP3R–Grp75–VDAC1 tethering complex. mTORC2/Akt/PACS2 signaling ensures MAM structural and functional wellbeing. mTORC2-driven Akt-mediated phosphorylation of IP3R3 fine-tunes Ca^2+^ release at the MAM. Hence, deficiency of mTORC2 disturbed MAM and enhanced mitochondrial calcium uptake. mTORC2-Akt-deficient cells suffer from an increased IMM potential and thereby defective mitochondrial functions and cellular homeostasis [91]. Inhibition of mTORC1 by rapamycin results in improved ER–mitochondria membrane coupling and thus mitochondrial Ca^2+^ uptake [152]. Epithelial cellular activities are highly regulated by the contact to the extracellular matrix (ECM). Hence, separation from the ECM will result in energy deprivation and metabolic stress in the cell. In such a condition, PERK will come into action and phosphorylate AMPK. Activated AMPK phosphorylates mTOR and induces autophagy to optimize energy utilization [153].

### Mitogen-activated protein kinase (MAPK)

MAPK is a Ser/Thr kinase that plays a role in cell death, inflammation, and cell proliferation [154]. MAPK or extracellular signal-regulated protein kinase 2 (ERK2) regulates cellular activities such as cell growth, division, differentiation, and cell death in response to cytokines and growth hormones. ERK 1/2, p38, JNK, and ERK5 are the subcategories of MAPK. MAPKs are capable of interacting with OMM and entering the mitochondrial lumen. In turn, this regulates ROS signaling, Ca^2+^ signaling, and thereby cell survival [155]. Cadmium is a heavy-metal pollutant that causes pulmonary ailments. The concentration of phosphorylated JNK, ERK, and p38 was high during treatment with cadmium in human bronchial epithelial cells, which indicates activation of MAPK signaling. Correspondingly, ROS accumulation and reduced mitochondrial membrane potential and viability were observed in treated cells [156].

## Pathological implications of kinases

Phosphorylation is an important posttranslational modification that determines the functionality of a protein. Any alteration in kinases can be manifested as abnormalities. A plethora of studies have been done regarding the pathological implications of kinases, which are responsible for diseases including cancers, autoimmune diseases, CVDs, inflammatory diseases, and diabetes. Since MAM is a major subcellular domain enriched with protein interactions, the dysregulated kinase activities underlie the cause of various diseases (Table 2).

### Protein kinase A (PKA)

PKA-dependent inhibitory phosphorylation of Drp1 abrogates mitochondrial fission and attenuates cardiomyocyte hypertrophy and hence heart growth [157]. Silencing of PKA in cardiomyocytes resulted in improper myocardial development, cardiac failure, and embryonic death [158]. Recently, the recuperative effect of melatonin was noticed in a mice disease model. Melatonin is capable of activating the MT2/cAMP/PKA/IRE1 signaling cascade (MT2: melatonin receptor type 1B- a G-protein coupled receptor (GPCR)) and thus reduces the ER stress and iron overload and reverses ferritin transport anomalies. Melatonin holds promising drug potential against ferroptosis complications in NAFLD [159]. Ferroptosis is lipid-peroxidation-dependent cell death and can be beneficial in the case of chemotherapy for glioblastoma. A combination drug, haloperidol with temozolomide, has an antagonizing effect on dopamine D2 receptor GPCR signaling. It causes upregulated cAMP/PKA signaling and thereby induces ER stress, autophagy, and ferroptosis. Altogether, it provides an antiproliferative effect in cancer and holds the prospect of a better future treatment intervention [160]. Liu et al. identified AKAP1/PKA/Grp75 as a novel drug target axis in oncocytes to improve their response to ferroptosis. AKAP1-anchored PKA becomes activated and Grp75 translocates from mitochondria to the MAM during lipid peroxidation in the ER. Grp75 activates the Nrf2 transcription factor upon PKA-mediated phosphorylation. Following that, Nrf2 activates a group of anti-ferroptotic genes and protects cancer cells against ferroptosis [161]. The imbalance in mitochondrial fission and fusion is an acceleratory factor in cancer progression. Chronic abundance of Cav-1 was observed in highly metastatic breast cancer cells. It is responsible for mitochondrial fission, ROS generation, and promoting metastasis via lamellipodia formation. Cav-1 can act as an anchoring protein for PKA and inhibit PKA activity. On the other hand, downregulation of Cav-1 resulted in mitochondrial redistribution of Mfn2 and Drp1, facilitating inhibitory phosphorylation of Drp1 by PKA and causing mitochondrial elongation [162]. A study conducted by Zhang et al. in mouse oocytes revealed the significance of cAMP/PKA activity in advanced maternal age-related oocyte quality deterioration due to the accumulation of ROS, oxidative stress, DNA damage, and apoptosis of immature oocytes. Treatment with C-natriuretic peptide (CNP), a potential meiotic inhibitor, increased the cAMP concentration and thereby the PKA activity. Increased PKA activity suppressed the mitochondrial recruitment of Parkin and thereby PINK1/Parkin-mediated mitophagy. Hence, amelioration of cAMP/PKA signaling could be an alternative clinical approach to avoid oxidative damage in aged oocytes [163].

### Protein kinase B (Akt)

In healthy cardiomyocytes, insulin activates PI3K/Akt signaling and glucose transporter type 4 (GLUT4) translocation to the cell membrane to induce glucose uptake. Insulin resistance is a leading cause of cardiomyopathies by reduced GLUT4 translocation, free fatty acid deposition, and cardiac impairment via altered PI3K-Akt-GLUT4 signaling pathways [164]. PI3K/Akt signaling is responsible for maintaining blood glucose homeostasis. Mice with null Akt2 displayed insulin resistance and a diabetic phenotype due to defective hormone actions on skeletal muscle and hepatic cells [165]. Disruption of the PI3K/Akt signaling pathway and abnormal expression of Akt2 have been recognized as a frequent event in many human malignancies such as ovarian cancer and breast cancer [166]. Aberrant Akt1 activity resulting from the negative regulator of Akt, inactive phosphatase and tensin homolog (PTEN), leads to cancer development [167]. Loss of the PTEN tumor suppressor activates the anti-apoptotic PI3K/Akt pathway and accelerates tumor growth [168]. Remarkably higher expression of Akt3 mRNA was observed in triple-negative breast cancer [169]. The rate of glycolysis is escalated in cancer cells owing to Akt-mediated phosphorylation of VDAC1. This enables efflux of hexokinase 2 from mitochondria and thereby glucose phosphorylation [91, 170]. The upregulation of glycolysis in tumor cells facilitates rapid proliferation, known as the Warburg effect [171].

### Polo-like kinase (PLK)

PLK2 was reported as a tumor suppressor in hematopoietic and lymphoid tissue-related carcinomas and solid tumors in organs [98]. Malfunctions of PLKs have been implicated in cancer and PD. The dysregulated Miro–Polo axis is involved in mitochondrial Ca^2+^ imbalance, mitochondrial dysfunction, and apoptosis, and finally, this can be causative for various diseases [96]. Interestingly, PLK mRNA is absent in healthy nondividing cells [172]. The posttranslational modifications of the protein synuclein are pivotal in synucleinopathies such as PD, AD, and Lewy body diseases. The phosphorylation of α-syn and β-syn at S129 and S118, respectively, is carried out by PLK1, 2, and 3. Further research is required to learn more about the impact and clinical benefits of these phosphorylation events [31].

### Phosphoinositol 3-kinase complex (PI3K)

PI3K/Akt signaling is vital for regulating the immune system and inflammation by mediating the activation of downstream signaling proteins. Any kind of assault on the PI3K/Akt pathway can lead to rheumatoid arthritis, leukemia, lymphoma, and other types of malignancies. PTEN inhibits the PI3K/Akt/mTOR pathway and mediates the autophagy of platelets and is a characteristic feature of an autoimmune disorder known as immune thrombocytopenia (ITP). In patients with ITP, longer platelet life spans and reduced apoptosis were observed. Owing to the increased concentration of PI3K, the phosphorylation of Akt and mTOR was found to be higher in patients with ITP. This increases their susceptibility to another autoimmune disorder, systemic lupus erythematosus (SLE). Sustained PI3K activation was found to be a causative factor in the development of SLE by downregulating PTEN [173]. According to studies, tumor cells, pancreatic stellate cells (PSCs), and tumor-infiltrating inflammatory cells show greater presence of activated PI3K family. The PI3K pathway and effective inflammatory conditions result from chemokines and cytokine secretions by immune cells at tumor sites [174]. PI3K–Akt–mTOR is a prominent signaling cascade leading to different malignancies such as breast, liver, colorectal, prostate, and gastric cancers [175]. Nrf-2 is a regulator of the antioxidant stress pathway and participates in the metabolic reprogramming of oncocytes. PI3K/Nrf-2 interaction provides a major proliferative signal in tumor tissues and cancer resistance. Nrf-2 makes cancer cells capable of thriving under stress conditions such as hypoxia, nutrient deprivation, and ER stress, hence this interaction could be a potential drug target [23]. JT003 is a synthetic adiponectin agonist against receptors AdipoR1 and AdipoR2 and has proved effective in reducing the activation of hepatic stellate cells in non-alcoholic steatohepatitis (NASH) and liver fibrosis. AdipoR signaling is important to enhance fatty acid oxidation and glucose uptake. The activation of hepatic stellate cells results in augmented fatty acid synthesis and uptake, oxidative stress, and ER stress and ends up in liver fibrosis. JT003 is effective in suppressing insulin resistance and lipogenesis by the PI3K/Akt/peroxisome proliferator-activated receptor γ (PPARγ) signaling pathway [176].

### Inositol-requiring kinase 1 (IRE1α)

Studies in triple-negative breast cancer cells revealed IRE1α-associated changes in lipid profiling and metabolism. IRE1α causes changes in lipid metabolism-related genes and accumulation of triacylglycerols. The higher presence of IRE1α contributes to an environment for cancer progression. The diacylglycerol acyltransferase 2 (DGAT2) mRNA encodes a key rate-limiting enzyme in triacylglycerol synthesis and is one of the targets of RIDD signaling. This suggests that ablation of IRE1α can result in the accumulation of TAG and accelerated cancerous growth. This study showed that this condition can be reversed by treatment with the DGAT2 inhibitor PF-06424439 (cat. no. HY-108341) [177]. The use of methamphetamine in patients with human immunodeficiency virus (HIV) exacerbates the complications of HIV-1-associated neurocognitive disorders by altering the metabolic activities of astrocytes. In the study, overexpression of IRE1α was noticed and resulted in increased mitochondrial respiration, glycolytic rates without proportionate ATP production, glutamic uptake, and inflammatory responses in astrocytes. This study reveals the curative efficacy of IRE1α by improving astrocyte functions for neuronal health in patients with HIV [178]. The IRE1α-Xbp-1 pathway directs the differentiation of adipocytes, neuronal cells, plasma cells, and osteoblasts. Regression due to differentiation of neuroblastoma cells is a clinical practice in the case of neuroblastoma, a common embryonic malignancy. Kaempferol (KFL) is a phytochemical with anticancerous properties capable of inducing the endoribonuclease activity of IRE1α. Treating neuroblastoma cells with KFL encouraged the differentiation of treated cells and holds great therapeutic promise against neuroblastomas [179]. Podocytes show high levels of autophagy compared with other cell types. Genetic silencing of IRE1α in glomerular epithelial cells exacerbates glomerular dysfunctions by impairing ER chaperone synthesis and autophagosome biogenesis since IRE1α stimulates the transcription of autophagosome genes such as Atg5 and Atg7 [180]. A study conducted in ovarian cancer patients revealed that the IRE1α–XBP1 signaling pathway is a promising clinical target to reestablish the T-cell metabolic homeostasis and antitumor properties. Malignant ascites fluid collected from ovarian cancer patients inhibited glucose uptake, causing nutrient deprivation and *N*-linked protein glycosylation defects in T-cells. This resulted in maladaptive IRE1α–XBP1-mediated suppression of mitochondrial activity and IFN-γ production. Activated XBP1 restricted the abundance of glutamine carriers and hence glutamine availability to maintain mitochondrial respiration in T-cells. The suppression of IRE1α-XBP1 signaling in T-cells in tumor tissues resulted in transcriptional modulation, improved antitumor efficacy, and increased overall survival [181].

### RNA-dependent protein kinase (PKR)-like ER kinase (PERK)

ER stress-induced, ROS-mediated apoptosis of cardiocytes leads to the clinical condition of diabetic cardiomyopathy. A distinguishable expression of PERK signaling and CHOP was found in cardiac cells suffering from hyperglycemia in mouse models. On the other hand, knockout of PERK provided an intensive protective effect against RO-mediated cell apoptosis [114]. Mfn2 has an inhibitory effect on PERK signaling and hence provides anti-apoptotic protection to podocytes in diabetic kidney diseases. The regulatory impact of Mfn2 on PERK can be utilized as a new therapeutic modality for diabetic nephropathy conditions [182]. Among the three UPR branches, overactivated PERK was noticed in the brain tissues of patients suffering from AD, PD, and dementia [183]. Apart from the actions of PERK, it is also implicated in age-related neurological disorders. Alterations in PERK expression can induce disturbed ROS exchange between ER and mitochondria by altering ER morphology. Studies in mice have shown that attenuation of PERK improved hypothalamus-dependent cognitive functions and reversed memory deficit [184]. Overexpression of PERK/eIF2α ceases protein synthesis, in turn resulting in synaptic failure and neuronal loss in the prion disease brain [185]. The same mechanism is the culprit behind FTD, in which PERK/eIF2α-mediated translational failure causes the formation of mutant Tau protein [186]. The sustained PERK-mediated UPR will result in increased expression of downstream factors such as ATF4 and CHOP and lead to AD. Recently, a study proved that reduction of ATF4 can ameliorate neuroinflammation and thus AD [187]. Fat deposition in hepatocytes induces ER stress, sustained activation of PERK and IRE1, and exacerbates hepatic steatosis. There was a reduction in PERK and IRE1 expression following the administration of diosgenin, an herbal steroidal saponin with a pharmaceutical effect on type 2 diabetes-associated NAFLD. Caspase 12 triggers ER stress-mediated apoptosis, and the application of diosgenin results in reduced expression of caspase 12 [188]. Phosphorylation of PERK and its downstream factors eIF2α and JNK that are involved in the ER-associated degradation pathway (ERAD) was notably reduced by JT003 in both NASH and liver fibrosis mouse models. This result suggests the recuperative effect of JT003 in regaining MAM homeostasis, thereby reducing cellular apoptosis in liver fibrosis [176]. Obesity is an accelerating factor of ER stress, oxidative stress, and inflammation. The condition activates PERK, and the PERK/SREBP-1c/FAS signaling pathway promotes lipogenesis (SREBP-1c: sterol regulatory element-binding protein-1c, FAS: fatty acid synthase). The Coix seed oil content in YYFZ powder can offer cardiovascular protection by reducing oxidative and ER stress and regaining MQC by suppressing the PERK/SREBP-1c/FAS signaling pathway [189].

### Leucine-rich repeat kinase 2 (LRRK2)

LRRK2 or PARK8 is a dominant, PD-associated gene that codes for a large and complex protein kinase abundant in the ER membrane [190]. Its protein product, dardarin, consists of a GTPase domain and a kinase domain [191]. Point mutations of LRRK2 are known to date and lead to sporadic and familial PD. LRRK2 G2019S is a common mutation with a gain-of-function effect that causes ER Ca^2+^ depletion and results in dysregulation of protein quality control. This leads to ER stress and cell death and is the cause of PD pathogenesis [120]. PD is one of the prevalent neurodegenerative conditions with an alarming accumulation of Lewy bodies or inclusion bodies causing the deliberate destruction of dopaminergic neurons in the substantia nigra [120, 192]. LRRK2 mutations result in the accumulation of inclusion substances and cell damage more than similar mutations in the paralog protein LRRK1 [192]. The findings on the deteriorative effect of LRRK2 are challenged by the study done by Yuan et al. in *Caenorhabditis elegans* and human neuroblastoma cells. They established that LRRK2 protects dopaminergic neurons against toxicity by the assemblage of α-synuclein, by upregulating translation of chaperone protein Grp78 [193]. The pathogenic mutation in LRRK2 (G2019S) disrupts the Miro removal and delays the initiation of mitophagy of damaged mitochondria. The degradation of the Miro protein is vital to detach mitochondria from the microtubule motors to make mitochondria static and available for mitophagy. The prolonged retention of Miro by mutant LRRK2 is a potential cause of familial and sporadic PD pathogenesis [194]. Mutation in the MAPT gene encoding Tau protein is detrimental to the nervous system by tangle formation. Cells with the pathogenic MAPT gene are defective in the recruitment of LRRK2 and Parkin to depolarized mitochondria and removal of Miro1 or Mfn2. The impairment of mitophagy causes the accumulation of damaged mitochondria. The study suggests that Tau may be necessary for the enzymatic activities of LRRK2 and Parkin and to execute Miro removal rather than their relocation to superfluous mitochondria [195]. Bardai et al. proved that either knockdown or overexpression of LRRK2 can lead to Tau-related neurotoxicity. Excessive expression of LRRK2 or mutant LRRK2 results in mitochondrial abnormalities by abnormal stabilization of filamentous actin and mislocalization of mitochondrial fission protein Drp1, and ends up with Tau neurotoxicity. Also, monomeric LRRK2 augments the Tau neurotoxicity, while expression of the wild form of LRRK2 or the mutant that enhances oligomerization of the protein can rescue tauopathy conditions [196].

### PTEN-induced kinase 1 (PINK1)

Mutation in PINK1 is a prevalent cause of early-onset autosomal recessive familial parkinsonism. PINK1 and Parkin show the loss-of-function mutation that results in mitochondrial transport as the key pathogenic event in sporadic and autosomal recessive PD [190]. Deficiency of mitochondrial PINK1 brought down the catalytic activity of complex 1 (NADH: ubiquinone oxidoreductase) activity, a fall in the electrochemical gradient, and reduced ATP production. Such cells are likely susceptible to Ca^2+^-dependent apoptotic stimuli and neuronal defects found in PD-related phenotypes [197]. Elevated expression of PINK1 was noticed in the white adipose tissues of mice fed with the high-fat diet. The disruption of brown adipose tissue caused by PINK1 deletion indicates that the regulation of mitophagy mediates the “whitening” of adipose tissue during the development of obesity [198]. The interaction between Parkin and PINK1 is essential in some neuropeptidergic neurons for the regulation of the sleep cycle. Enhanced ER–mitochondria connection and excessive lipid trafficking are due to the loss-of-function mutation of Parkin or PINK1. It caused the depletion of ER phosphatidylserine, which is essential for the production and secretion of neuropeptide-containing vesicles. Eventually, this manifested as a defect in morning anticipation and circadian rhythm, a premotor symptom in PD models [199]. Idiopathic pulmonary fibrosis is an age-related lung disorder. As age increases, alveolar cells are highly vulnerable to ER stress, which downregulates PINK1. Since PINK1 is a key regulator of MQC, the consequences that ensue include mitochondrial depolarization and dysfunction, increased apoptosis, and expression of proinflammatory and profibrotic factors. The young mouse model also showed manifestations of swollen and dysfunctional mitochondria and lung fibrosis upon lack of PINK1 [200].

### Glycogen synthase kinase-3β (GSK-3β)

Two proteins, namely fused in sarcoma (FUS) and TAR DNA binding protein-43 (TDP-43), are known to induce hyperactivation of GSK-3β and lead to amyotrophic lateral sclerosis (ALS) [201]. PI3K-PKB-dependent or antagonist-mediated inactivation of GSK-3β is found to be effective in reducing apoptosis and ischemic–reperfusion injury in cardiocytes [135]. GSK-3β phosphorylation or inactivation is a better way to inhibit mitochondrial permeability transition pore (MPTP) opening, which is a lethal risk of ischemic–reperfusion injury. The exact mechanism behind this postconditioning is not clear [202]. The inhibition of GSK-3β will be beneficial during myocardial ischemia–reperfusion therapy by reducing mitochondrial Ca^2+^ concentration and hence the susceptibility to myocardial cell death [136]. During a study in a T2DM mouse model, upregulated interaction between GSK-3β and Drp1 and effective alteration in mitochondrial fission and morphology were observed. It resulted in diabetes-associated synaptic injury and adverse effects on synaptic plasticity in hippocampus neurons. Treatment with 4-benzyl-2-methyl-1,2,4-thiadiazolidine-3,5-dione (TDZD8) induced p-Ser9- GSK-3β inactivation and significantly reduced diabetes-related synaptic anomalies [203]. Any mutations or overexpression of TDP‐43 activate GSK‐3β by reducing the inhibitory phosphorylation of Ser-9. In turn, GSK‐3β perturbs ER–mitochondria contacts and Ca^2+^ signaling by disrupting the interaction between VAPB and PTPIP51 [204], becoming a cause of ALS and FTD [201]. ALS mutations (FUS‐R521G) or overexpression of FUS also mediated activation of GSK-3β in the same manner as TDP-43 and disrupted ER–mitochondria contact [205]. GSK-3β is a promising drug target in clinical practice for AD and stroke. The immunoreactivity of GSK-3β and phosphorylated GSK-3β is found to be increased in neuronal tissues with age, and further investigation of this will provide insights regarding GSK-3β-related neurodegenerative diseases [206].

### Adenosine monophosphate-activated protein kinase (AMPKα)

Endothelial nitric oxide synthase (eNOS) is a downstream target of AMPK and is important for cardiac functions. Insulin resistance reduces the phosphorylation of AMPK and eNOS activity, resulting in reduced coronary blood flow and coronary heart diseases via the reduced AMPK–eNOS signaling pathway [164]. Previous studies have proved the reduced phosphorylation of AMPK as an underlying cause of many heart diseases in high-fat-diet mouse models [207, 208]. The expression level of FUNDC1 involved in MAM formation was found to be high in the kidney cells of diabetic mice with diabetic nephropathy. Treatment with capsaicin resulted in declining MAM formation in podocytes. Capsaicin enhanced the Ca^2+^ intake through transient receptor potential cation channel subfamily V member 1 (TRPV1), and it caused AMPK activation. AMPK reduced the transcription of FUNDC1 and thereby MAM formation [209]. A study in cardiomyocytes of newborn mice corroborated the inhibitory role of AMPK on FUNDC1. The cells with a low abundance of AMPK were exposed to hyperglycemia and induced FUNDC1-driven ER–mitochondria contacts and mitochondrial abnormalities, and ended up with cardiomyopathy. Hence, AMPK-induced FUNDC1 suppression is a promising treatment modality for diabetic cardiomyopathy [210]. Concomitantly, profound degradation of MAM and mitochondrial integrity was found in human and mouse kidney tissues grown under high-glucose conditions. The mentioned impacts are due to the activation of mitogen-activated protein kinase 1 (MAPK1) and subsequent loss of PACS-2, which is essential for MAM maintenance by preventing mitochondrial fragmentation. The MAPK1–PACS2 axis is rendered as a target of therapy for diabetic kidney diseases [211]. High glucose induces AMPK and MAPK1, both having opposite effects on MAM formation. The actual mechanism of regulation of MAM integrity due to hyperglycemia remains unclear. The inhibition of AMPK activity by saturated fatty acids reduces autophagy, induces ROS production, and results in the activation of inflammasomes. The inflammation in the blood stem cells can alter insulin signaling, thereby minimizing glucose tolerance, and leading to T2DM [143]. One of the major proteins involved in lipid metabolism is AMPK. Diosgenin administration is beneficial for relieving NAFLD by lowering the insulin resistance index, relieving hepatic lipid deposition, improving fatty acid beta-oxidation, and reducing de novo lipogenesis. The clinical effects of diosgenin are via AMPK-mediated regulation of the manifestation of the transcription factor sterol regulatory element binding protein-1 (SREBP1) and the gene coding acetyl CoA carboxylase [188]. The AMPK–Mfn2 axis was proven to enhance MAM functions and related phenotypes. The activation of the nonselective cation channel, transient receptor potential vanilloid type 1 (TRPV1), induces phosphorylation of AMPK and enhances the AMPK–Mfn2 axis. This resulted in enhanced MAM formation, Ca^2+^ transfer, ATP synthesis, and ameliorated myocardial hypertrophy [212]. JT003, a dual AdipoR agonist, was found to be effective in the treatment of NASH and liver fibrosis by suppressing fatty acid synthesis genes and augmenting fatty acid oxidation genes via activating the AMPK/PPARα signaling pathway. Besides, enhanced AMPK signaling ameliorates ER–mitochondria axis dysfunctions in NASH mouse models [176].

### Pyruvate dehydrogenase kinase 4 (PDK4)

Ablation of PDK4 in mice treated with a high-fat diet resulted in reduced MAM integrity, ER stress, and blood sugar, and improved insulin sensitivity and glucose tolerance [145, 213, 214]. Vascular calcification is the condition in which vascular smooth muscle cells become calcified, being observed in diabetes, atherosclerosis, and aging. It is noticed that PDK4 accelerates the process by direct phosphorylation of the Smad1/5/8 complex, which is instrumental for bone morphogenetic protein 2 (BMP2) signaling [215]. Mitochondria are the major sites for alcohol metabolism in hepatocytes. PDK4 phosphorylates the chaperone Grp75 at multiple sites, causing alcohol-mediated increased Ca^2+^ flux through IP3R. The mitochondrial Ca^2+^ overload resulted in mitochondrial aberration, which is instrumental to the pathogenesis of alcohol-associated liver disease. The inhibition or ablation of PDK4 has a protective effect against alcohol-associated liver disease [216].

### Casein kinase II (CK2)

The kinase CK2A1 phosphorylates PACS2 and facilitates Ca^2+^ flux through PKD2. Phospho-dead mutations in PACS2 affect the Ca^2+^ influx into mitochondria via PKD2 accumulation in the cytoplasm and cause exaggerated neurotransmitter release from glutamatergic neurons, eventually ending up with epileptic symptoms associated with DEE66 [9]. The hyperphosphorylation of Miga and related proteins by CKI and Ca^2+^/calmodulin-dependent protein kinase II (CaMKII) at different locations results in extra ERMCSs and neurodegenerative diseases such as AD [74]. FUNDC1-mediated mitophagy following cardiac ischemia injury is a rescue event to minimize the damage of mitochondria and related complexities. However, increased levels of CK2α induce phosphorylation-linked inhibition of FUNDC1 and arrest mitophagy of damaged mitochondria. This results in defects in mitochondrial biogenesis and the electron transport chain and favors cardiomyocyte dysfunction [217].

### Mammalian target of rapamycin (mTOR) kinase

Activation of AGC kinase members such as serum/glucocorticoid-regulated kinase 1 (SGK1), Akt, and PKC is regulated by mTOR, and these enzymes are relevant in case of apoptosis, cancer, and diabetes [150, 218, 219]. Many of the MAM proteins are known for their contribution to carcinogenesis by their anti-apoptotic or pro-apoptotic functions through manipulating the Ca^2+^ flux between the mitochondria and ER. Akt phosphorylates IP3R3 and blocks apoptosis by inhibiting IP3R3-mediated ER–mitochondria Ca^2+^ release. The activity of Akt is regulated by mTORC2 (activator) and PTEN and PML (inhibitors). PTEN is a dominant inhibitor of PI3K/Akt signaling pathways, and its loss causes constitutive activation of Akt in several cancer variants. The activity of PP2a is supported by PML and downregulates the Akt functions [220]. Cancer cells have the potential to attain a reversible “drug-tolerant persister” (DTP) state or diapause to evade chemotherapeutic stress. DTP state is a loophole for evading chemotherapy and a major hurdle in cancer treatment. Downregulation of the mTOR pathway was noticed in cancer cells and is responsible for the slow cell cycle, dormancy, and drug resistance [221]. Meanwhile, mTOR suppression brought autophagy into action, and autophagy has a key role in mediating the diapause state. The application of a combination of chemotherapy and autophagy inhibitors brings these cells out of the drug-tolerant state and causes robust cell death. This could be a new treatment modality for cancer [222]. The Ras family of G-proteins is vital for regulating various cellular signaling involving the PI3K/Akt/mTOR pathway, which is essential for normal cardiac growth. Studies have proved that overactivation of JNK and p38 signaling marks the onset of chronic heart diseases. Ras induces the overexpression of JNK and p38 signaling through the PI3K/AKT/mTOR pathway. YiYiFuZi powder (YYFZ) is a classical Chinese medicine beneficial for treating CVDs. Administration of YYFZ in a CVD model rat reduced the pathological symptoms, and the network pharmacology revealed that YYFZ acts through the Ras and PI3K/Akt/mTOR signaling pathways [223].

### Mitogen-activated protein kinase (MAPK)

The missense mutation in MAPK results in cancer-prone disorders and oncogenesis [224]. Overexpression or mutations of Ras protein lead to activation of MAPK. In turn, MAPK promotes Drp1-mediated mitochondrial fission and hence tumor phenotype. The Ras–ERK2 axis should be evaluated as a clinical target in cancer therapy [225]. The MAPK pathway takes part in the phosphorylation of Tau protein. Hence, an impaired MAPK pathway can result in brain damage in AD patients [154]. Quercetin is a drug with anti-inflammatory and anti-oxidant properties. A study carried out by Guoxiu Zu et al. suggested quercetin as a potential drug candidate against AD and T2DM by targeting the MAPK signaling pathway [154].

## Kinases as therapeutic targets

The design and development of potential kinase inhibitors is an attractive area of medical research to combat various kinase abnormalities (Table 3). Reperfusion therapy is a promising clinical intervention against CVDs. Paradoxically, the same causes myocardial injury as well. The reperfusion injury salvage kinase (RISK) pathway is a cascade process that offers cardioprotection by reducing infarct size, by either activating Akt or arresting GSK3β [226]. Maintaining Cav-1 at a phosphorylated state by enhancing local kinase activity would be helpful to reduce mitophagy and metastasis, hence being applicable during cancer chemotherapy [71]. Small inhibitory molecules known as kinase-inhibiting RNase attenuators (KIRAs) offer modulation of IRE1α activities and have dual benefits such as induction of XBP1 splicing and inhibition of insulin mRNA decay. The wise application of KIRAs opens avenues for targeted therapy of ER stress-related diseases such as diabetes [110]. Sepsis is the condition of systemic inflammation in the body following infections. Encephalopathy is one of the complications found in septic patients. The activation of glucagon-like peptide-1 receptor (GLP-1R) in microglial cells by liraglutide has beneficial effects such as alleviating ER stress, inflammatory responses, and apoptosis. This is achieved by the manifestation of survival-related proteins by the cAMP/PKA/CREB pathway (CREB: cAMP response element-binding protein) [227]. In the case of Akt, six types of inhibitors are in clinical trials based on their mode of action. ATP-competitive inhibitors, lipid-based Akt inhibitors, pseudo-substrate inhibitors, allosteric inhibitors, antibodies, and inhibitors interact with the PH domain of Akt [228]. Prolonged ER stress switches on extensive PERK activation during UPR and results in degenerative disorders. Treatment with the compound trazodone was found to be an effective inhibitor of PERK in a prion disease model [183]. The FDA has accepted 37 kinase inhibitors as clinical drugs against lung and breast cancers.

**Table 3:** Diseases associated with kinase malfunctioning and kinase inhibitors in clinical trials

Kinases	Malfunction/disease	Inhibitors/drugs	Ref.
PKA	Cardiomyocyte hypertrophy	H89 (*N*-[2-*p*-bromocinnamylamino-ethyl]-5-isoquinolinesulfonamide)	[157, 234]
Akt/PKB	Breast cancer, ovarian cancer, diabetes, insulin resistance	Ipatasertib, capivasertib, afuresertib	[90, 165]
PLK	Cancer	ZK-thiazolidinone, NMS-1, CYC-800, DAP-81, LC-445, BI-2536, BI-6727	[172]
PI3K	Inflammation, pancreatic cancer, cervical cancer	Thymoquinone, alpelisib, copanlisib, idelalisib, duvelisib, umbralisib	[99, 174, 235, 236]
IRE1α	T2DM, cancer, obesity	Sunitinib, toyocamycin	[237]
PERK	T2DM, FTD, Wolcott–Rallison syndrome (WRS)	HC-5404, GSK2606414, GSK2656157	[183, 186, 238, 239]
LRRK2	PD	Abivertinib	[120, 240]
PINK1	PD	Phenolic acids from *Eucommia ulmoides* Oliver (EuO) leaves	[241]
GSK-3β	T2DM, synaptic injury in the hippocampus	TDZD8	[203]
AMPK	Diabetic nephropathy, diabetic cardiopathy, D-NAFLD	Capsaicin, DIO	[188, 209]
PDK4	Obesity, insulin resistance	Diisopropylamine dichloroacetate	[242]
CK2	DEE-66	Quercetin, quinalizarin	[9, 243]
mTOR	Cancer, T2DM	Rapamycin	[150, 218, 219]
MAPK1	Cancer	U0126	[244]

## Conclusions

The MAM is a protein interaction arena between the ER and mitochondria. MAM proteins are involved in various physiological functions such as tethering (Mfn2, Fis1, and BAP31), antitethering (TpMs), apoptosis (Fis1 and BAP31), mitochondrial fission (Fis1 and Drp1), and Ca^2+^ oscillations (IP3R, Grp75, VDAC, and VAPB). Any alteration in protein interactions can result in disease phenotypes including AD, PD, diabetes, ALS, obesity, CVDs, malignancies, and NAFLD. There are still debatable opinions regarding MAM protein functions, such as the tethering and antitethering functions of Mfn2. Kinases act as important structural and functional MAM modulators, playing roles behind the scenes by carrying out protein phosphorylation events. The kinases function in a cascade manner and are interconnected in various cellular activities. Mostly, kinase mutations result in inappropriate physiological actions. However, in the case of LRRK2, mutants that encourage oligomerization of the kinase diminish the chances of Tau pathology. Different agonist and antagonist molecules are found to modulate kinase actions and are now being studied to correct disease-prone protein crosstalks.

MAM-related studies could result in several future medical developments. The stabilization of the MAM thickness in neuron axons can moderate the Aβ level and mitochondrial axonal transport. This could be an effective clinical approach for conditions such as AD. Over 25 kinase inhibitors have been approved as drugs to treat malignancies. Understanding the cancer-related pathway of MAM kinases could lead to the development of novel kinase-inhibitory drug molecules that disrupt tumor growth. The development of an effective method to isolate pure MAM from a cell is highly demanded to facilitate the proteomic analysis of MAM and might help to provide a clear picture of cell conditions and help prognosis. The elucidation of the connection between MAM proteins and glucose metabolism will help to target the pathway to improve insulin resistance, diabetes, and obesity conditions. Since MAM is cell specific, future research could offer personalized clinical strategies focusing on the MAM proteome. The development of well-equipped imaging will facilitate the screening of MAM interactions in real time and in living cells. The development of MAM-associated biomarkers would be useful for early detection and monitoring of disease progression.

There are some limitations in the field of MAM since it is an emerging area of research. Several key proteins in MAM have been studied, yet the complete protein composition of MAMs remains uncertain. The MAM proteome is cell specific, and since it is a dynamic structure, the protein composition can vary according to cellular needs. Molecular signaling and other factors that regulate the composition and plasticity of the MAM according to various physiological and pathological conditions must be studied. The regulation of tethering to determine the extent of MAM in various cells has to be explained. MAM dysfunctions are manifested as diseases including neurodegeneration, diabetes, cancers, and CVDs. However, it is still unclear whether MAM disruption is a cause or consequence of these diseases. Since MAM is emerging as a clinical target, targeting MAM selectively without affecting normal cell functions is demanding more research. Since the MAM is a dynamic structure, the biochemical basis behind context-based kinase recruitment to the MAM is still unclear. The mitochondria have extra genetic material within them. The role of mitochondrial DNA in the synthesis of MAM proteins and kinases has to be studied. Physical factors and conditions behind the activation or inactivation of kinases have to be elucidated. Several small kinase inhibitory molecules are in clinical trials as drug candidates. The drugs have to be designed in such a way that they target only specific MAM proteins without any nontarget toxicities.

A major challenge in kinase-targeting therapeutics is the presence of structurally related kinases, hence the effects of kinase inhibitors are not confined to intended targets. Recent studies have reported that several FDA-approved kinase inhibitors used to treat cancer can also reverse diabetes. Kinases acquire inhibitor resistance via mutations within gatekeeper residues that occur during chemotherapy. Hence, the pathology and treatment for malignancies become more complex. During cancer therapy, blocking multiple kinases may be required to obtain the desired outcome. In such a scenario, combinations of kinase inhibitors are used as drugs. Since the kinase inhibitor targets can be present in various tissues in the human body, the reliability of kinase inhibitor drugs is questioned by their off-target toxicities. More sophisticated therapeutic techniques have to be executed to overcome the above-mentioned challenges.

This review recapitulates recent research, research trends, research gaps, and potential directions for future studies in the field of the ER–mitochondria axis and MAM kinases. Future research has to be directed to address the aforementioned gaps, limitations, and challenges to establish effective MAM-targeted treatments. Combined in silico and in vitro studies will help to extend our knowledge of the MAM and the related kinome and to translate laboratory findings into clinical practice for human wellbeing.

## Data Availability

Not applicable.

## Abbreviations

MAM: Mitochondria-associated membranes
ER: Endoplasmic reticulum
ER stress: Endoplasmic reticulum stress
MCS: Membrane contact sites
PM: Plasma membrane
ERMES: ER–mitochondria encounter structure complex
OMM: Outer mitochondrial membrane
T2DM: Type 2 diabetes mellitus
FDA: Food and Drug Administration of the USA
MQC: Mitochondrial quality control
PERK: RNA-dependent protein kinase (PKR)-like ER kinase
ROS: Reactive oxygen species
UPR: Unfolded protein responses
SERCA: Sarcoplasmic/endoplasmic reticulum Ca ATPase-2 (SERCA) pump
PINK1: PTEN induced kinase 1
GSK-3β: Glycogen synthase kinase‐3β
FUS: Fused in sarcoma
TDP-43: TAR DNA binding protein-43
ALS: Amyotrophic lateral sclerosis
AD: Alzheimer’s disease
CVDs: Cardiovascular diseases
PD: Parkinson’s disease
PLK1: Polo-like kinase 1
FTD: Frontotemporal dementia
Mfn2: Mitofusin 2
BiP: Binding immunoglobulin protein
PACS2: Phosphofurin acidic cluster sorting protein 2
TpMs: Trichoplein/mitostatin
CMT2A: Charcot–Marie–Tooth disease type 2A
Fis1: Fission protein 1 homolog
ERAD: Endoplasmic reticulum-associated degradation
BAP31: B-cell receptor-associated protein 31
Akt/PKB: Protein kinase B
PI3K: Phosphoinositide 3-kinase
PML: Promyelocytic leukemia
BECN1: Beclin-1
Drp1: Dynamin-related protein 1
VAPB: Vesicle-associated membrane protein-associated protein B
PTPIP51: Protein tyrosine phosphatase-interacting protein-51
IP3R: Inositol 1,4,5-triphosphate receptors
Grp75: Glucose-regulated protein 75
VDAC1: Voltage-dependent anion channel 1
Sig-1R: Sigma-1 receptor
FUNDC1: FUN14 domain containing protein 1
Ero1α: ER oxidoreductin 1α
REEP1: Receptor expression-enhancing protein-1
Cav-1: Caveolin-1
PKA: Protein kinase A
AKAPs: A-kinase anchoring proteins
RyRs: Ryanodine receptors
cAMP: Cyclic adenosine monophosphate
PKB/Akt: Protein kinase B
PACS 2: Phosphofurin acidic cluster sorting protein 2
PP2a: Protein phosphatase 2a
mTOR: Mammalian target of rapamycin kinase
PI3KC3: Class 3 PI3K
PAS: Pre-autophagosomal structure
IRE1α: Inositol-requiring kinase 1
E-Syt1: Extended-synaptotagmin 1
Xbp1: X-box binding protein 1
RIDD: Regulated IRE1-dependent decay
CASP2: Caspase-2
JNK: C-Jun N-terminal kinase
ORMDL3: Orosomucoid-like protein 3/ORMDL sphingolipid biosynthesis regulator 3
TRAF2: TNF receptor-associated factor 2
EIF2AK3: Eukaryotic translation initiation factor 2α kinase 3
eIF2α: Eukaryotic transcriptional initiation factor-α subunit
CHOP: C/EBP homologous protein
LRRK2: Leucine-rich repeat kinase 2
DMiro: *Drosophila* Miro
AMPK: Adenosine monophosphate-activated protein kinase
Mff: Mitochondrial fission factor
ULK1: UNC-51-like kinase
PDK: Pyruvate dehydrogenase kinase
CK2: Casein kinase II
MAPK1: Mitogen-activated protein kinase 1
ERK2: Extracellular signal-regulated protein kinase 2
NAFLD: Nonalcoholic fatty liver disease
GPCR: G-protein coupled receptor
PTEN: Phosphatase and tensin homolog
ITP: Immune thrombocytopenia
DGAT2: Diacylglycerol acyltransferase 2
DEE66: Developmental and epileptic encephalopathy-66
CaMKII: Ca^2+^/calmodulin-dependent protein kinase II
DTP: Drug-tolerant persister
KIRAS: Kinase-inhibiting RNase attenuators
CREB: Cyclic adenosine monophosphate response element-binding protein
